# A novel function for the transcription factor sensitive to proton rhizotoxicity1 in promoting anthocyanin accumulation in strawberry

**DOI:** 10.1111/pbi.70194

**Published:** 2025-06-10

**Authors:** Ruiqing Bian, Jinxiang Yao, Yuxin Nie, Yuying Zhang, Zhengjia Wu, Junxiang Zhang, Zhihong Zhang

**Affiliations:** ^1^ Liaoning Key Laboratory of Strawberry Breeding and Cultivation, College of Horticulture Shenyang Agricultural University Shenyang China

**Keywords:** FvSTOP1, FvMYB1, FvTT19, strawberry, anthocyanin

## Abstract

Sensitive to proton rhizotoxicity1 (STOP1), a C2H2‐type zinc finger transcription factor, has been widely studied as a core transcription factor in adapting plants to diverse environmental stresses. However, whether STOP1 regulates anthocyanin accumulation in plants is still unknown. This study identified the FvSTOP1 in woodland strawberry. FvSTOP1 is a nuclear‐localized protein with self‐activation activity and has the highest expression in the ripening fruit and root. Stable overexpression and knockout of *FvSTOP1* in woodland strawberries promoted and reduced anthocyanin accumulation in fruits. FvSTOP1 activated the expression of glutathione *S*‐transferase *FvTT19* to promote anthocyanin accumulation. Interestingly, FvSTOP1 interacted with the strawberry anthocyanin repressor FvMYB1, but FvSTOP1 did not affect the protein abundance of FvMYB1. Furthermore, FvSTOP1 also interacted with the strawberry anthocyanin activator FvbHLH33, forming the FvSTOP1–FvMYB1–FvbHLH33 complex. FvSTOP1 reduced the repression of FvMYB1–FvbHLH33 on the expression of *FvTT19*, *FvCHS*, and *FvF3H* via obstructing the formation of the repression complex FvMYB1–FvbHLH33 to promote anthocyanin accumulation. Together, FvSTOP1 promoted anthocyanin accumulation by activating the expression of *FvTT19* and hampering the formation of the FvMYB1–FvbHLH33 repression complex in strawberry fruits. This study identifies the novel function of STOP1 in plant species and will lay a foundation for improving the colour quality of strawberry fruits.

## Introduction

Strawberries are widely cultivated worldwide because of their high nutritional and economic value (Li *et al*., 2023). Fruit colour is an important agronomic trait of strawberries, and the diversity of colours in strawberries is attributed to various levels of anthocyanin accumulation (Kosar *et al*., 2004). Anthocyanins are important secondary plant metabolites (You *et al*., 2023). They are accountable for the flowers and fruits blue, purple, or red, improving plant resistance to various adverse environments and pollination and seed dispersal via attracting insects and animals (Allan *et al*., 2008; Wen *et al*., 2020). Moreover, anthocyanin‐rich foods benefit humans as antioxidants, reducing the onset of various chronic diseases such as cancer, obesity, and cardiovascular disease (Giampieri *et al*., 2014; Zhang *et al*., 2014).

Anthocyanin biosynthesis starts with phenylalanine precursor and needs a series of enzymes including phenylalanine ammonia‐lyase (PAL), chalcone synthase (CHS), flavonoid 3‐hydroxylase (F3H), dihydroflavonol‐4‐reductase (DFR), anthocyanidin synthase (ANS), and several glycosyltransferases (UFGTs) (Xu *et al*., 2015; Yue *et al*., 2023; Zhang *et al*., 2020). Anthocyanin biosynthetic genes are mainly regulated by MYB, bHLH, and WD40 transcription factors, which act independently or form MYB/bHLH/WD40 (MBW) complex to carry into effect (Gonzalez *et al*., 2008; Xu *et al*., 2015). The strawberry FaMYB10/FaMYB5‐FabHLH3/FabHLH33‐FaTTG1 complex promotes anthocyanins' biosynthesis (Hossain *et al*., 2018; Lin‐Wang *et al*., 2014). Strawberry FaMYB1 interacts with two bHLHs (AN1 and JAF13) to inhibit anthocyanin biosynthesis (Aharoni *et al*., 2001). Additionally, the FaMYB9/FaMYB11–FabHLH3–FaTTG1 complex in strawberries specifically regulates proanthocyanins' biosynthesis (Schaart *et al*., 2012).

Anthocyanins are produced in the endoplasmic reticulum (ER) and then transported to vacuoles for storage, which confers plants with different colours (Grotewold, 2004; Pourcel *et al*., 2010). Anthocyanins are transported through membrane vesicle‐mediated and membrane transporter‐mediated transport (Zhao and Dixon, 2010). The main transporters of anthocyanins include MATE, multidrug resistance‐associated protein (MRP), and glutathione *S*‐transferase (GST) (Zhao and Dixon, 2010). The MATE‐mediated anthocyanin transport depends on the reverse transport of H^+^/Na^+^. MATE transports carry anthocyanins into the vacuole driven by H^+^/Na^+^ with ATP and pump protons out of the vacuole (Marinova *et al*., 2007). Plant MATE transporters play an important role in primary and secondary metabolisms, nutrient homeostasis, stress responses, and hormone transport (Wang *et al*., 2017). MRPs are one of the transmembrane transporters' ATP‐binding cassette (ABC) superfamily. To date, only some members of the MRP‐type ABC transporter proteins have been identified as participating in the transmembrane transport of flavonoids on the vacuolar membrane (Zhao *et al*., 2011). After the biosynthesis of anthocyanins in the cytoplasm, GST‐catalysed glutathione (GSH) directly covalently binds to anthocyanins forming glutathione‐conjugates and then recognized by the MRPs on the vacuole membrane, which leads to the glutathione‐conjugates transmembrane transport into vacuoles. Hence, MRP proteins have been identified as transmembrane transport anthocyanins into vacuoles (Ishikawa *et al*., 1997). Most GSTs are distributed in the cytoplasm, with a few distributed in chloroplasts and nuclei (Dixon *et al*., 2010). The maize (*Zea mays*) *ZmBz2* gene encodes a type III GST and is the earliest discovered *GST* gene related to anthocyanin transport. ZmBz2 catalyses the conjugation of GSH to cyanidin 3‐*O*‐glucoside (C3G) which is transported into the vacuole via a glutathione pump (GS‐X pump) on the vacuolar membrane (Alfenito *et al*., 1998). GST involved in anthocyanin transport has been widely identified in crops, such as *Transparent testa 19* (*AtTT19*) in Arabidopsis (Kitamura *et al*., 2004; Sun *et al*., 2012), *VvGST1* and *VvGST4* in grape (*Vitis vinifera*) (Conn *et al*., 2008), *Anthocyanin 9* (*PhAn9*) in petunia (*Petunia hybrida*) (Mueller *et al*., 2000), and *Reduced Anthocyanins in Petioles* (*RAP*) in woodland strawberry (*Fragaria vesca*) (Luo *et al*., 2018). In conclusion, MATE, MRP, and GST are important proteins involved in the transport of plant anthocyanins.

Some transcription factors regulating anthocyanin transport genes have been identified. VvMYBPA1 and VvMYBPA2 from grape (*Vitis vinifera*) promote proanthocyanidins (PA) accumulation by inducing the expression of *VvMATE1* (Bogs *et al*., 2007; Terrier *et al*., 2009). Chickpeas CaFUSCA3 containing a B3 domain affects proanthocyanidin accumulation in the seed coat by binding to the promoter of *CaMATE23* (Thakro *et al*., 2023). Peach PpMYB10.1 promotes anthocyanin accumulation by directly binding to the promoter of *PpABCC1* (Sylvia *et al*., 2023). The *FvABCC8* promoter was activated by FvMYB10, FvbHLH33, and FvMYC1 to improve anthocyanin accumulation in woodland strawberry (Qian *et al*., 2023). Similarly, many other MYBs promoting anthocyanin accumulation by activating the expression of genes involved in anthocyanin transport have been identified, including MdMYB1 from apple (*Malus domestica*) (Jiang *et al*., 2019), RsMYB1a from radish (*Raphanus sativus*) (Lai *et al*., 2021), AcMYB110 from kiwifruit (*Actinidia chinensis*) (Liu *et al*., 2019), and LcMYB1 from litchi (*Litchi chinensis*) (Hu *et al*., 2016), and FvMYB10 from woodland strawberry (Luo *et al*., 2018). Taken together, most transcription factors modulating genes participating in anthocyanin transport are MYBs, and other transcription factors are rarely reported.

Sensitive to proton rhizotoxicity1 (STOP1) is a C2H2‐type (Cys‐Cys‐His‐His motif) zinc finger transcription factor with four zinc finger (ZFs, ZF1–ZF4) domains (Iuchi *et al*., 2008). The ZF1, ZF2, and ZF4 domains belong to the C2H2‐type, whereas ZF3 belongs to the C2HC‐type or the C2H2‐type (Iuchi *et al*., 2007). *Arabidopsis* STOP1 enhances aluminium tolerance by regulating the expression of aluminium tolerant genes *Aluminium‐activated malate transporter* (*ALMT1*), *multidrug and toxic compound extrusion* (*MATE1*) (Liu *et al*., 2009), *Aluminium‐sensitive 3* (*ALS3*) (Sawaki *et al*., 2009), *RNA export factor1* (*RAE1*) (Zhang *et al*., 2019), and *Glutamate‐dehydrogenase1/2* (*GDH1/2*) (Liu *et al*., 2009; Sawaki *et al*., 2009; Tokizawa *et al*., 2021). STOP1 has also been identified to regulate self‐phosphorylation and stability, thereby enhancing aluminium resistance in *Arabidopsis* (Fan *et al*., 2016; Zhou *et al*., 2023). Overexpression of *MtSTOP1* upregulates the expression of *MtMATE66* and *MtMATE69* of *Medicago truncatula* to induce the transport of citric acid, which alters the iron homeostasis and results in iron deficiency chlorosis of seedlings (Wang *et al*., 2017). AtSTOP1 induces the expression of *AtALMT1* to promote malic acid binding more Fe^3+^, thereby facilitating lateral root formation in plants under low phosphorus conditions (Mora‐Macías *et al*., 2017). AtSTOP1 directly binds to the promoter of *Nitrate transporter 1.1* (*NTR1.1*) to activate its expression at low pH conditions, thereby enhancing plant nitrate uptake, improving nitrogen utilization efficiency, and creating a favourable rhizospheric pH for root growth (Ye *et al*., 2021). In addition, STOP1 binds to the promoter of *CBL interacting protein kinase 23* (*CIPK23*) to promote its expression, which enhances K^+^ transport in plant roots and improves salt and drought tolerance of *Arabidopsis* (Sadhukhan *et al*., 2019). *Arabidopsis* STOP1 controls the expression of *Heat shock factor A2* (*HsfA2*) and enhances low oxygen tolerance (Enomoto *et al*., 2019). In conclusion, STOP1 plays a vital role in aluminium resistance, root growth, and abiotic stress, but it is unclear whether STOP1 modulates anthocyanin accumulation in plants.

This study identified a new anthocyanin regulator, C2H2‐type zinc finger transcription factor FvSTOP1, in woodland strawberry. Stable overexpression and gene editing indicated that FvSTOP1 positively regulated the anthocyanin accumulation in strawberry fruits. Moreover, FvSTOP1 promoted anthocyanin accumulation by activating the expression of *FvTT19* and interfering with the formation of the FvMYB1–FvbHLH33 repression complex of anthocyanins in strawberry fruits. This study reveals the mechanism of FvSTOP1 in strawberry promoting anthocyanin accumulation, expands the function of STOP1 in plants, and lays the foundation for improving strawberry quality.

## Results

### FvSTOP1 is a transcription factor with four zinc finger domains

To identify novel transcription factors regulating anthocyanin accumulation in strawberry, a yeast two‐hybrid (Y2H) screen was conducted using the master transcriptional repressor FvMYB1 (Aharoni *et al*., 2001; File S1) as bait. This screening revealed an interaction between FvMYB1 and FvSTOP1. While STOP1 has been widely characterized as a core transcription factor enabling plant adaptation to environmental stresses, its potential role in anthocyanin biosynthesis remains unexplored. These findings suggest that FvSTOP1 may function as a novel regulator of anthocyanin accumulation in strawberry (Nie *et al*., 2025). To investigate the function of strawberry FvSTOP1, the coding region of FvSTOP1 was cloned from woodland strawberry ‘Ruegen’. The coding region of *FvSTOP1* is 1572 bp (File S1), encoding 523 amino acids. Phylogenetic trees between FvSTOP1 and its homologues showed that FvSTOP1 had the closest evolutionary relationship with rose RcSTOP1 (*Rosa chinensis*) (Figure S1a). DNAMAN software was used to analyse the amino acid sequences. The similarity of amino acid sequences between FvSTOP1 and RcSTOP1 was 84.89%, while the similarity of amino acid sequences between FvSTOP1 and model plants *Arabidopsis* AtSTOP1 was 52.95%. In addition, FvSTOP1 had four conserved C2H2‐type (Cys–Cys–His–His) zinc finger domains, which were similar to its homologues. The positions of the four zinc finger domains in FvSTOP1 were 271–291 aa, 320–351 aa, 358–378 aa, and 385–408 aa, respectively (Figure S1b). The amino acid sequence alignment indicated that FvSTOP1 was a C2H2‐type zinc finger transcription factor with four zinc finger domains.

### FvSTOP1 is a nuclear‐localized protein with self‐activating activity and the highest expression in the root and ripening fruit

**Figure 1:**
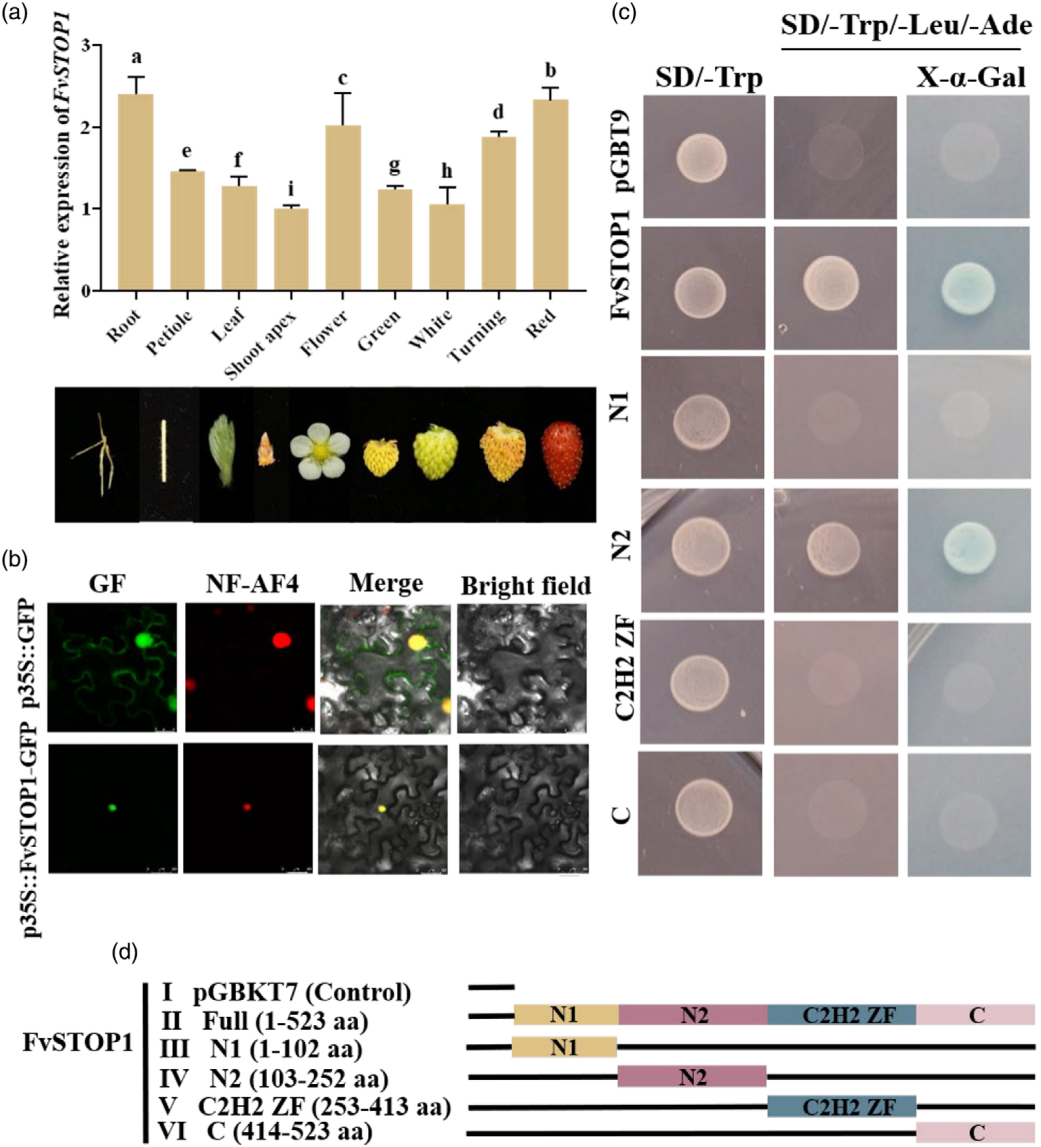
Expression patterns, subcellular localization, and self‐activation activity of FvSTOP1. (a) RT‐qPCR detected expression patterns of *FvSTOP1* in different organs. Different letters indicate significant differences compared with the shoot apex. Data are the mean ± SD of three biological replicates with Tukey's hoc test (*P* < 0.05). (b) Subcellular localization of FvSTOP1. *p35S*::GFP and *p35S*::FvSTOP1‐GFP were infiltrated into the leaves of 4‐week‐old tobacco, respectively. NF‐AF4 is a nuclear localization protein marker. Bar of NF‐AF4 = 25 μm, Scale bar of FvSTOP1 = 50 μm. (c) Self‐activation activity of *FvSTOP1*. BD‐FvSTOP1 was transformed into Y2H gold cells, and detected on agar plates (SD/−Trp and SD/−Trp/−Leu/−Ade/X‐α‐gal). (d) The segments of the FvSTOP1 protein, N1 (1–102 aa), N2 (103–252 aa), C2H2 ZF (253–413 aa), C (414–523 aa).

The RT‐qPCR was conducted to detect the expression level of the *FvSTOP1* gene in different organs of woodland strawberry ‘Ruegen’ including root, petiole, leaf, shoot apex, flower, and fruits in four developmental stages (green, white, turning, and red). The highest expression level of *FvSTOP1* was in the root and the lowest was in the shoot apex (Figure 1a). The expression of *FvSTOP1* in the root was 2.4 times higher than that in the shoot apex. During fruit development, the transcript level of *FvSTOP1* was significantly increased in the turning and ripening fruit compared with the green and white fruits (Figure 1a), indicating that *FvSTOP1* probably plays a role in fruit ripening. To investigate the subcellular distribution of FvSTOP1, the fusion plasmid *p35S*::FvSTOP1‐GFP was constructed and injected into 4‐week‐old leaves of tobacco (*Nicotiana benthamiana*). GFP controls without FvSTOP1 showed fluorescent signals in both the cytoplasm and nucleus, while the fluorescence signals of *p35S*::FvSTOP1‐GFP only showed in the nucleus (Figure 1b).

To detect the self‐activation activity of FvSTOP1, the fusion plasmid FvSTOP1‐pGBT9 was constructed and transferred into yeast‐competent cells. pGBT9 contains the GAL4 DNA‐binding domain. The protein was fused with GAL4‐pGBT9 and then transformed into the yeast‐competent cells. If the protein has self‐activating activity, it will activate the expression of the reporter gene in yeast, thereby enabling it to grow on a defective medium (SD/−Trp/−Leu/−Ade). The negative control was the pGBT9 empty vector. Yeast cells harbouring the complete coding region of pGBT9‐FvSTOP1 grew well on the defective agar plates (SD/−Trp and SD/−Trp/−Leu/−Ade). On the defective agar plates with alpha‐galactosidase (SD/−Trp and SD/−Trp/−Leu/−Ade/X‐α‐gal), the yeast cells displayed blue, which demonstrated that FvSTOP1 activates alpha‐galactosidase (Figure 1c). To further clarify which regions of FvSTOP1 have self‐activation activity, we divided the complete coding region of FvSTOP1 into four independent regions based on structural domains: N1 (1–102 aa), N2 (103–252 aa), C2H2 ZF (253–413 aa), and C (414–523 aa) (Figure 1d) to perform self‐activation analysis. The yeast cells containing the N2 region grew well on the defective agar plates (SD/−Trp and SD/−Trp/−Leu/−Ade), while the yeast cells harbouring N1, C2H2 ZF, and C regions did not grow. Together, these results suggested that *FvSTOP1* was highly expressed in the root and ripening fruit, localized in the nucleus, and had self‐activation activity in the N2 region.

### FvSTOP1 promotes anthocyanin accumulation in strawberry fruits

**Figure 2:**
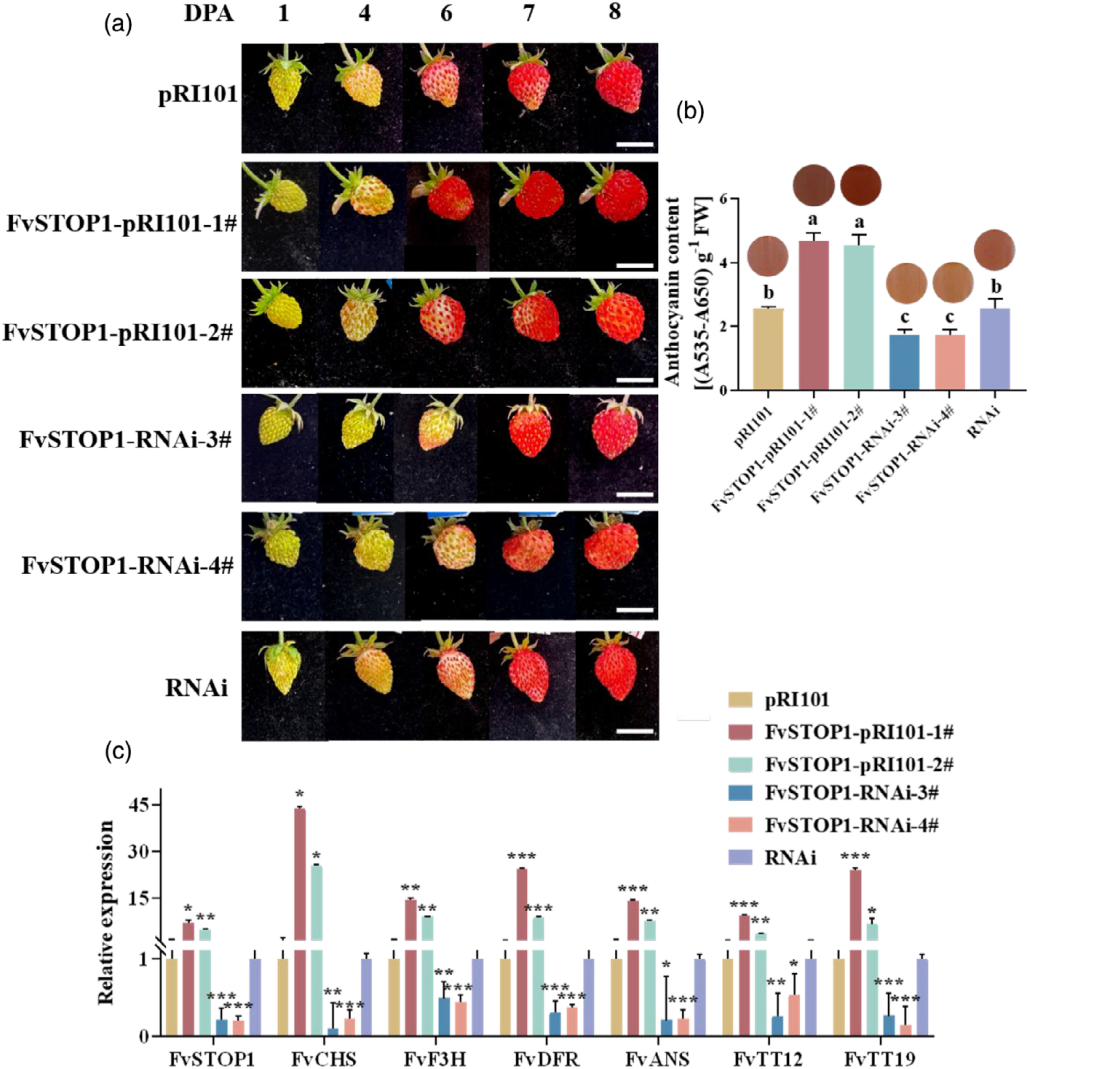
Transient overexpressing and silencing of *FvSTOP1* in woodland strawberry fruits. (a) Phenotypes of transient overexpressing and silencing of *FvSTOP1* fruits. *p35S*::*FvSTOP1* and *FvSTOP1‐*RNAi plasmids were injected into the fruits of the ‘Ruegen’ at the big green stage (18 days after pollination), and at least 15 fruits were selected for transient assays. Scale bar = 1 cm. (b) Anthocyanin contents in transient overexpressing and silencing of *FvSTOP1* fruits. The circles represent the colour of the anthocyanin extract. FW is fresh weight. Different letters represent significant differences compared with the control with Tukey's hoc test (*P* < 0.05). (c) Relative expression of anthocyanin‐related genes in transient overexpressing and silencing of *FvSTOP1* fruits. Data are the mean ± SD of three biological replicates. Significant differences were analysed by Student's *t*‐test (*P < 0.05, **P < 0.01, ***P < 0.001).

To detect the function of the strawberry *FvSTOP1* gene, we constructed the overexpression vector of *FvSTOP1* (*p35S*::*FvSTOP1*) and the silencing vector of *FvSTOP1* (*FvSTOP1*‐RNAi) and transiently transformed them into strawberry fruits at the big green stage (18 days after pollination). Surprisingly, we found that overexpressing *FvSTOP1* accelerated fruit ripening, and the anthocyanin content of the fruits of overexpressing *FvSTOP1* was 1.81 times higher than that of the control (Figure 2a,b). Conversely, silencing *FvSTOP1* delayed fruit ripening, and the anthocyanin content of the fruits of silencing *FvSTOP1* decreased by 31.97% compared with the control (Figure 2a,b). We further analysed the expression of anthocyanin biosynthetic genes *FvCHS*, *FvF3H*, *FvDFR*, *FvANS*, and anthocyanin transport genes *FvTT12* and *FvTT19*. The expression of anthocyanin‐related genes in the fruits of overexpressing *FvSTOP1* was significantly increased, while the expression of anthocyanin‐related genes in the fruits of silencing *FvSTOP1* was significantly decreased relative to the control (Figure 2c). This information suggested that *FvSTOP1* played a vital role in anthocyanin accumulation in strawberry fruits.

**Figure 3:**
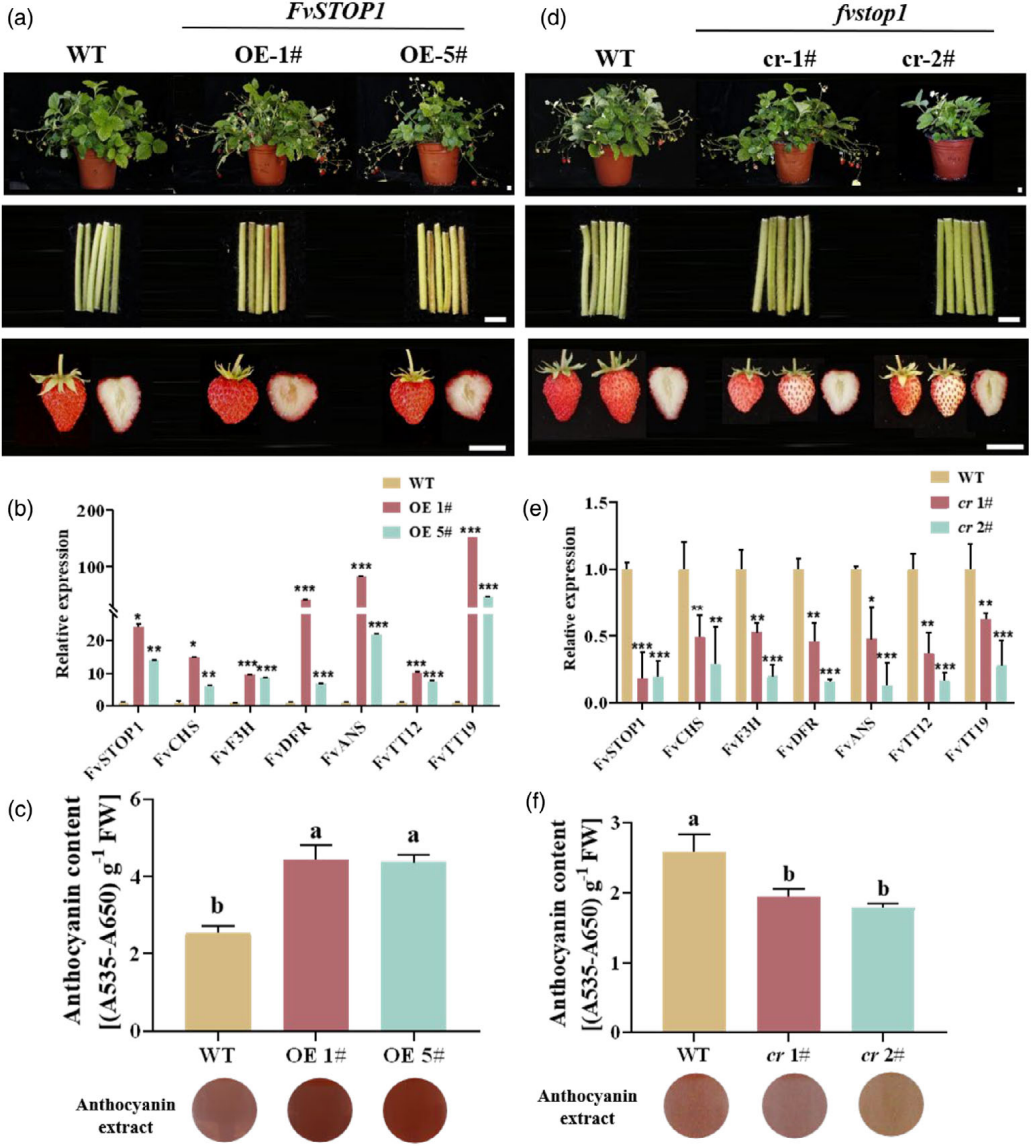
Stable overexpressing and knockout *FvSTOP1* in strawberry. (a) Phenotypes of wild‐type (‘Ruegen’, WT) and overexpressing *FvSTOP1* (*FvSTOP1*‐OE) plants, petioles, and fruits. Scale bar = 1 cm. (b) Relative expression of anthocyanin‐related genes in wild‐type (‘Ruegen’, WT) and overexpressing *FvSTOP1* plant fruits (*FvSTOP1*‐OE). (c) Anthocyanin contents in wild‐type (‘Ruegen’, WT) and overexpressing *FvSTOP1* plant fruits (*FvSTOP1*‐OE). The circles represent the colour of the anthocyanin extract. FW is fresh weight. (d) Phenotypes of wild‐type (‘Ruegen’, WT) and knockout *FvSTOP1* (*fvstop1‐cr*) plants, petioles, and fruits. Scale bar = 1 cm. (e) Relative expression of anthocyanin‐related genes in wild‐type (‘Ruegen’, WT) and *fvstop1‐cr* fruits. (f) Anthocyanin contents in wild‐type (‘Ruegen’, WT) and *fvstop1‐cr* fruits. The circles represent the colour of the anthocyanin extract. FW is fresh weight. Data are the mean ± SD of three biological replicates. Different letters represent significant differences compared with the control with Tukey's hoc test (*P* < 0.05). Significant differences were analysed by Student's *t*‐test (*P < 0.05, **P < 0.01, ***P < 0.001).

To further verify the function of *FvSTOP1*, we obtained stable overexpression of *FvSTOP1* transgenic plants by *Agrobacterium tumefaciens‐*mediated transformation (Figure 3a). The transcript level of *FvSTOP1* in overexpression of *FvSTOP1* transgenic plants was verified via RT‐qPCR, and the relative expression of *FvSTOP1* was significantly increased in overexpressing *FvSTOP1* lines (*FvSTOP1*‐OE 1#, 5#) (Figure 3b). Compared with the wild‐type control (WT), the petiole and fruit colour of *FvSTOP1‐*OE lines were redder, but there was no remarkable difference in the leaves and flowers (Figures 3a and S2a). The anthocyanin content of *FvSTOP1*‐OE mature fruits was significantly increased compared with the WT plants. The anthocyanin content of *FvSTOP1*‐OE #1 and #5 increased by 1.75 and 1.73 times compared with WT, respectively (Figure 3c). Next, we measured the transcript levels of anthocyanin‐related genes in overexpressing *FvSTOP1* transgenic plants. Compared with the WT, the expression of anthocyanin‐related genes, such as *FvCHS*, *FvF3H*, *FvDFR*, *FvANS*, *FvTT12*, and *FvTT19* in overexpressing *FvSTOP1* plants was greatly increased, especially *FvTT19* (Figure 3b). These results demonstrated that *FvSTOP1* positively regulated anthocyanin accumulation in strawberry.

In addition, we obtained two stable *FvSTOP1* knockout transgenic lines (*fvstop1‐cr* 1#, 2#) using CRISPR/cas9 gene editing technology. We designed two sgRNAs with different target sequences to *FvSTOP1*. Two independent homozygous mutant lines (*fvstop1‐cr* 1#, 3#) were identified in the T1 generation. *fvstop1‐cr* 1# had a 295 bp inversion between target 1 and target 2 (Figure S3a,b). *fvstop1‐cr* 3# had a 1 bp insertion in target 1 forming a stop codon TGA and a 1 bp deletion in target 2, respectively (Figure S3e,f). The *fvstop1‐cr* 3# line had an obvious delayed growth and development (Figure S4). Therefore, we hybridized *fvstop1‐cr* 1# and *fvstop1‐cr* 3# to obtain the biallelic mutant *fvstop1‐cr* 2# (Figure S3c,d). The fruit colour of *fvstop1‐cr* lines (1# and 2#) became light red compared with WT, while the petiole, flower, and leaves had no significance relative to WT (Figures 3d and S2b). The content of anthocyanin in the fruits of *fvstop1‐cr* lines (1# and 2#) was significantly lower than that in WT fruits, decreasing by 25.0% and 30.7% (Figure 3f). Subsequently, the transcript levels of anthocyanin‐related genes in knockout transgenic plants of *FvSTOP1* were measured. The transcript levels of anthocyanin‐related genes, such as *FvCHS*, *FvF3H*, *FvDFR*, *FvANS*, *FvTT12*, and *FvTT19* in knockout *FvSTOP1* plants were significantly decreased (Figure 3e). Thus, strawberry FvSTOP1 promoted anthocyanin accumulation was closely related to the expression of anthocyanin‐related genes.

To investigate flavonoid metabolic alterations beyond anthocyanins in transgenic plants, we conducted metabolomic profiling of WT and *FvSTOP1* knockout lines (*fvstop1‐cr* 1# and 2#). A total of 169 flavonoid metabolites were identified between WT and the two knockout transgenic lines (Figure S5a–d). Differential analysis using *P*‐value < 0.05 and |log_2_FC| > 0.58 thresholds revealed distinct regulation patterns: WT versus *fvstop1‐cr* 1# displayed eight significantly up‐regulated and ten down‐regulated metabolites, whereas WT versus *fvstop1‐cr* 2# showed six up‐regulated and 54 down‐regulated metabolites (Figure S5b,d). The Venn diagram showed 12 common differentially expressed substances between WT and the two knockout lines (two were up‐regulated and ten were down‐regulated) (Figure S5e). Among them, eight flavones and flavonols (chrysoeriol‐7‐*O*‐rutinoside, neodiosmin, oroxin B, kaempferol‐7‐*O*‐rhamnoside, luteolin‐*O*‐malonylhexoside, selgin‐*O*‐malonylhexoside, 3,7‐Di‐*O*‐methylquercetin, lonicerin), one chalcone and dihydrochalcone (naringenin), one isoflavone (maackiain), one flavanone (eriodictyol‐*O*‐malonylhexoside) and one anthocyanin (Cyanidin‐3‐*O*‐rutinoside). These data demonstrate that FvSTOP1 regulates biosynthesis across multiple flavonoid subclasses beyond anthocyanins (Figure S5f,g).

### FvSTOP1 directly binds to the promoter of *FvTT19* to promote anthocyanin accumulation

**Figure 4:**
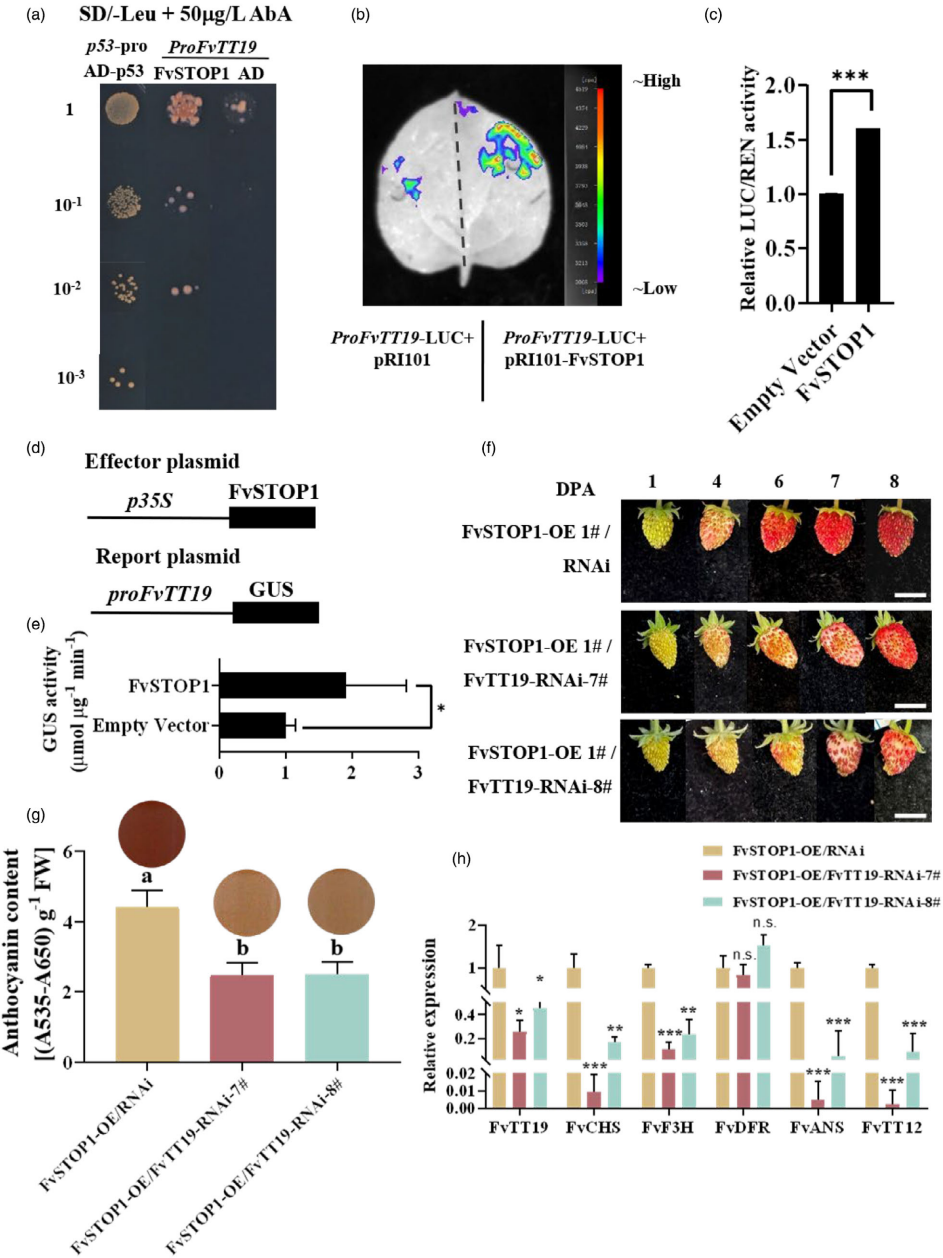
FvSTOP1 binds to the promoter of *FvTT19* to promote anthocyanin accumulation. (a) Yeast one‐hybrid (Y1H) assay displaying the binding of FvSTOP1 to the promoter of *FvTT19*. The transformed Y1H gold cells were grown on SD/−Leu agar plates containing Aureobasidin A (AbA = 50 μg/L). The negative control was *proTT19*‐pAbAi + AD. The positive control was p53‐promoter + AD‐p53. (b) Luciferase (LUC) assay showed that FvSTOP1 promoted the promoter activity of *FvTT19* in tobacco. (c) Relative LUC/REN values of the binding between FvSTOP1 and *FvTT19* promoter in tobacco. (d) GUS assay demonstrated that FvSTOP1 activated the expression of the *FvTT19* in strawberry. (e) GUS activity detection revealed that FvSTOP1 promoted the promoter activity of *FvTT19* in strawberry. The empty vector was pRI101. The experimental group was (FvSTOP1‐pRI101 + *proFvTT19*‐GUS). The control group was (pRI101 + *proFvTT19*‐GUS). (f) The transient assay was conducted in overexpressing *FvSTOP*1 plant fruits (*FvSTOP1*‐OE) to verify the direct binding between FvSTOP1 and *FvTT19* promoter. Injection of *FvTT19*‐RNAi into *FvSTOP1*‐OE fruits (18 days after pollination). At least 15 fruits were selected for transient assays. Scale bar = 1 cm. (g) Anthocyanin contents in transient transformation fruits (*FvTT19*‐RNAi injected into *FvSTOP1*‐OE fruits). The circles represent the colour of the anthocyanin extract. FW, fresh weight. (h) Relative expression of anthocyanin‐related genes in transient transformation fruits (*FvTT19*‐RNAi injected into *FvSTOP1*‐OE fruits). Data are the mean ± SD of three biological replicates. Different letters represent significant differences compared with the control with Tukey's hoc test (*P* < 0.05) and Student's *t*‐test (*P < 0.05, **P < 0.01, ***P < 0.001).

To explore whether FvSTOP1 promotes anthocyanin accumulation by directly regulating the expression of anthocyanin‐related genes, we detected the binding of FvSTOP1 to the promoters of *FvCHS*, *FvF3H*, *FvDFR*, *FvANS*, *FvTT12*, and *FvTT19* by yeast one‐hybrid (Y1H) assay. We found that only the third region of the *FvTT19* promoter (−534 to −1 bp from ATG) grew well on SD/−Leu with AbA (50 μg/L) agar plates compared with the control (Figure 4a). However, the yeast containing the promoters of *FvCHS*, *FvF3H*, *FvDFR*, *FvANS*, and *FvTT12* could not grow well on SD/−Leu agar plates with different AbA concentrations compared with the control (Figure S6), suggesting that FvSTOP1 could directly bind to the promoter of *FvTT19*.

To detect the regulatory effect of FvSTOP1 on *FvTT19*, we first ligated the promoter of *FvTT19* to the luciferase (LUC) reporter gene (*proFvTT19*‐LUC) and co‐injected it with the effector plasmid (*p35S*::*FvSTOP1*) into tobacco leaves for the luciferase assay. FvSTOP1 had an activation effect on *FvTT19*, and the LUC activity co‐injection of *proFvTT19*‐LUC + *p35S*::*FvSTOP1* was 1.61 times higher than that of the control (Figure 4b,c). We further ligated the promoter of *FvTT19* to the GUS reporter gene (*proFvTT19*‐GUS) and co‐injected it with the effector plasmid (*p35S*::*FvSTOP1*) into strawberry fruits and *p35S*::*pRI101* without FvSTOP1 as an empty vector for the GUS activity assay. Similar to the LUC activity, the GUS activity of the co‐injection of *proFvTT19*‐GUS + *p35S*::*FvSTOP1* was 1.91 times higher than that of *proFvTT19*‐GUS + pRI101 (Figure 4d,e). These results suggested that FvSTOP1 promoted the expression of *FvTT19* by regulating the promoter of *FvTT19*.

Furthermore, we tested the function of *FvTT19* in strawberry. We first detected the expression of *FvTT19* in different organs of ‘Ruegen’. The transcript abundance of *FvTT19* gradually increased during fruit development, reaching the highest in mature fruit. The lowest transcript level of *FvTT19* was identified in the shoot apex (Figure S7). Next, we constructed the overexpression vector of *FvTT19* (*p35S::FvTT19*) and the silencing vector of *FvTT19* (*FvTT19*‐RNAi) to investigate the function of *FvTT19* by *Agrobacterium tumefaciens*‐mediated transient transformation assays. We found that overexpression of *FvTT19* accelerated fruit ripening and the colour of the fruit turned dark red compared with the control, while silencing *FvTT19* delayed fruit ripening and fruit colour was light red relative to the control (Figure S8a). The content of anthocyanin in overexpressing *FvTT19* fruits was 1.80 times higher than that of the control, whereas silencing *FvTT19* fruits decreased by 43.0% compared with the control (Figure S8b). The relative expression of anthocyanin‐related genes in the fruits of overexpressing and silencing *FvTT19* was significantly up‐regulated and down‐regulated relative to the control, respectively (Figure S8c). These results showed that strawberry *FvTT19* played a positive regulatory role in anthocyanin accumulation.

To further examine the direct regulation of *FvTT19* by FvSTOP1 in strawberry, we conducted transient transformation assays in overexpressing *FvSTOP1* plants (*FvSTOP1*‐OE). The *Agrobacterium* containing silencing *FvTT19* plasmid was injected into the fruits of the *FvSTOP1*‐OE plant (*FvSTOP1*‐OE/*FvTT19*‐RNAi) at the big green fruit stage (18 days after pollination). Compared with the control (*FvSTOP1*‐OE/RNAi), the ripening of the fruit was remarkably delayed after the injection of silencing *FvTT19* bacterial suspension into the fruits of *FvSTOP1*‐OE plants, and the fruit colour was light red (Figure 4f). About 8 days later, the anthocyanin content of the fruits of *FvSTOP1*‐OE plants injected with *FvTT19*‐RNAi bacterial suspension was reduced by 43.7% compared with the control (Figure 4g). These results further demonstrated that FvSTOP1 enhances the expression of *FvTT19*, thereby promoting the accumulation of anthocyanins. We also tested the expression of anthocyanidin‐related genes in transient transformation strawberry fruits. The transcription levels of *FvCHS*, *FvF3H*, *FvDFR*, *FvANS*, and *FvTT12* in the fruits of *FvSTOP1*‐OE plants injected with *FvTT19*‐RNAi were significantly decreased compared with the control (Figure 4h). In summary, FvSTOP1 directly bound the promoter of *FvTT19* to improve its expression, thereby promoting anthocyanin accumulation in strawberry fruits.

### FvSTOP1 interacts with FvMYB1 *in vitro* and *in vivo*

To further elucidate the mechanism by which FvSTOP1 regulates anthocyanin accumulation in strawberry, we performed a screen to identify anthocyanin‐associated transcription factors interacting with FvSTOP1. Notably, our prior Y2H screen using FvMYB1 as bait had already revealed a physical interaction between FvMYB1 and FvSTOP1 (Nie *et al*., 2025).

**Figure 5:**
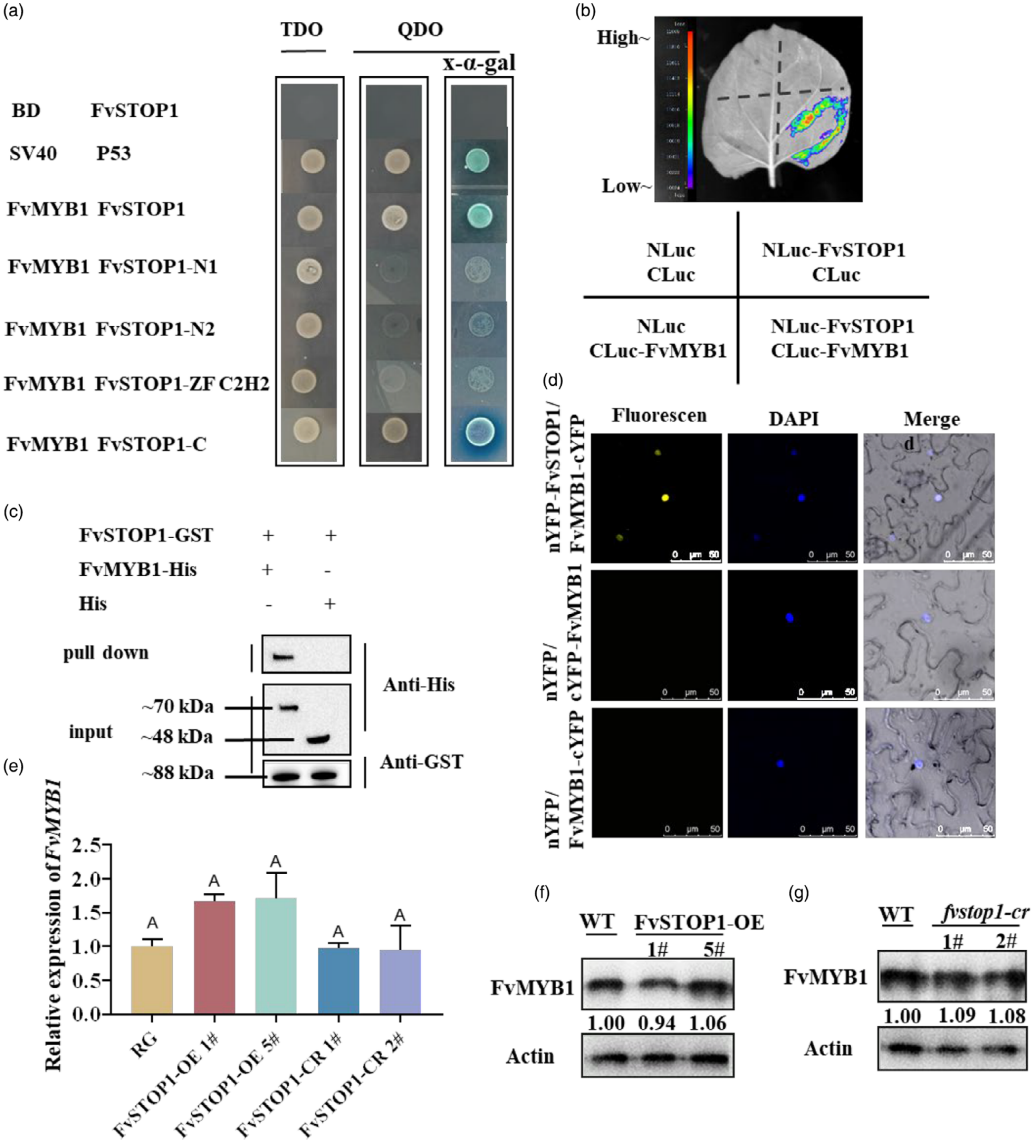
Analysis of the interaction between FvSTOP1 and FvMYB1 and the interaction on the effect of the transcript and protein levels of FvMYB1. (a) Yeast two‐hybrid (Y2H) assay detected the interaction between FvSTOP1 and FvMYB1. The negative control was the co‐transformation of BD and FvSTOP1‐AD. The positive control was the co‐transformation of SV40‐BD and P53‐AD. (b) Luciferase complementation imaging (LCI) assay revealed the interaction between FvSTOP1 and FvMYB1. (c) Pull‐down assays demonstrated the interaction between FvSTOP1 and FvMYB1. The protein band emphasized by the anti‐His illustrates that FvSTOP1‐GST pulled down FvMYB1‐His protein. (d) Bimolecular fluorescence complementation (BiFC) assay revealed the interaction between FvSTOP1 and FvMYB1. Bar = 50 μm. DAPI (4′,6‐Diamidino‐2′‐phenylindole) is a blue nuclear dye. (e) Relative expression levels of *FvMYB1* in the fruit of *FvSTOP1*‐OE and *fvstop1*‐cr transgenic plants. Different letters represent significant differences compared with the control with Tukey's hoc test (*P* < 0.05). (f) The relative abundance of FvMYB1 protein in the fruit of *FvSTOP1*‐OE transgenic plants using the FvMYB1 antibody. (g) The relative abundance of FvMYB1 protein in the fruit of *fvstop1*‐cr transgenic plants using the FvMYB1 antibody.

To confirm that FvSTOP1 interacted with FvMYB1, we initially used a Y2H assay. Yeast harbouring FvMYB1‐pGBT9 and FvSTOP1‐pGADT7 grew well on defective agar plates (SD/−Trp/−Leu/−Ade/−His), indicating that FvSTOP1 interacted with FvMYB1 (Figure 5a). We further detected which region of the FvSTOP1 protein interacted with FvMYB1. We found that the C‐terminal of FvSTOP1 interacted with FvMYB1, while the N‐terminal and the C2H2 ZF‐terminal of FvSTOP1 did not interact with FvMYB1 (Figure 5a). Next, a luciferase complementation imaging (LCI) assay was conducted in tobacco leaves to detect whether FvSTOP1 interacts with FvMYB1. The co‐injection area of NLuc‐FvSTOP1 and FvMYB1‐CLuc has an intense fluorescence signal, while no signals were observed in NLuc and FvMYB1‐CLuc or NLuc‐FvSTOP1 and CLuc (Figure 5b). In addition, we also conducted a pull‐down assay to examine whether FvSTOP1 interacts with FvMYB1. The fusion protein FvMYB1‐His and pCold‐TF (His) were incubated with FvSTOP1‐GST, respectively. Immunoblotting with anti‐His and anti‐GST showed that FvMYB1‐His could be pulled down, while the pCold‐TF (His) as a negative control could not (Figure 5c). Finally, the bimolecular fluorescence complementation (BiFC) assay illustrated that FvSTOP1 interacted with FvMYB1. We observed the YFP fluorescence signals in the nucleus during the co‐expression of FvMYB1‐cYFP and nYFP‐FvSTOP1, while the control group (cYFP + nYFP‐FvSTOP1, FvMYB1‐cYFP + nYFP) had no YFP fluorescence signal (Figure 5d). The above results suggested that FvSTOP1 interacted with FvMYB1 *in vitro* and *in vivo*.

### FvSTOP–FvMYB1 interaction does not affect the transcript and protein levels of FvMYB1

To verify whether FvSTOP1 interacted with FvMYB1 affects the transcript level of the *FvMYB1* gene and the protein abundance of FvMYB1, the transcript level and protein abundance of FvMYB1 in overexpressing and knockout *FvSTOP1* plants were analysed by RT‐qPCR and Western blot (WB) analysis, respectively. Compared with the WT (woodland strawberry, ‘Ruegen’) plants, there was no significant difference in the transcript levels of *FvMYB1* in overexpressing and knockout *FvSTOP1* plants (Figure 5e). Furthermore, no significant difference in the protein abundance of FvMYB1 in overexpressing and knockout *FvSTOP1* plants was found compared with the WT plants (Figure 5f,g). The above results suggested that the interaction between FvSTOP1 and FvMYB1 did not affect the transcript and protein level of FvMYB1 in strawberry.

### FvSTOP1 interacts with FvbHLH33 protein *in vitro* and *in vivo*

The strawberry R2R3‐type MYB1 usually interacts with bHLH33 to co‐regulate the expression of anthocyanin biosynthetic genes (Aharoni *et al*., 2001). To detect whether FvSTOP1 interacts with FvbHLH33 (File S1) besides FvMYB1, we verified the interaction between FvSTOP1 and FvbHLH33.

**Figure 6:**
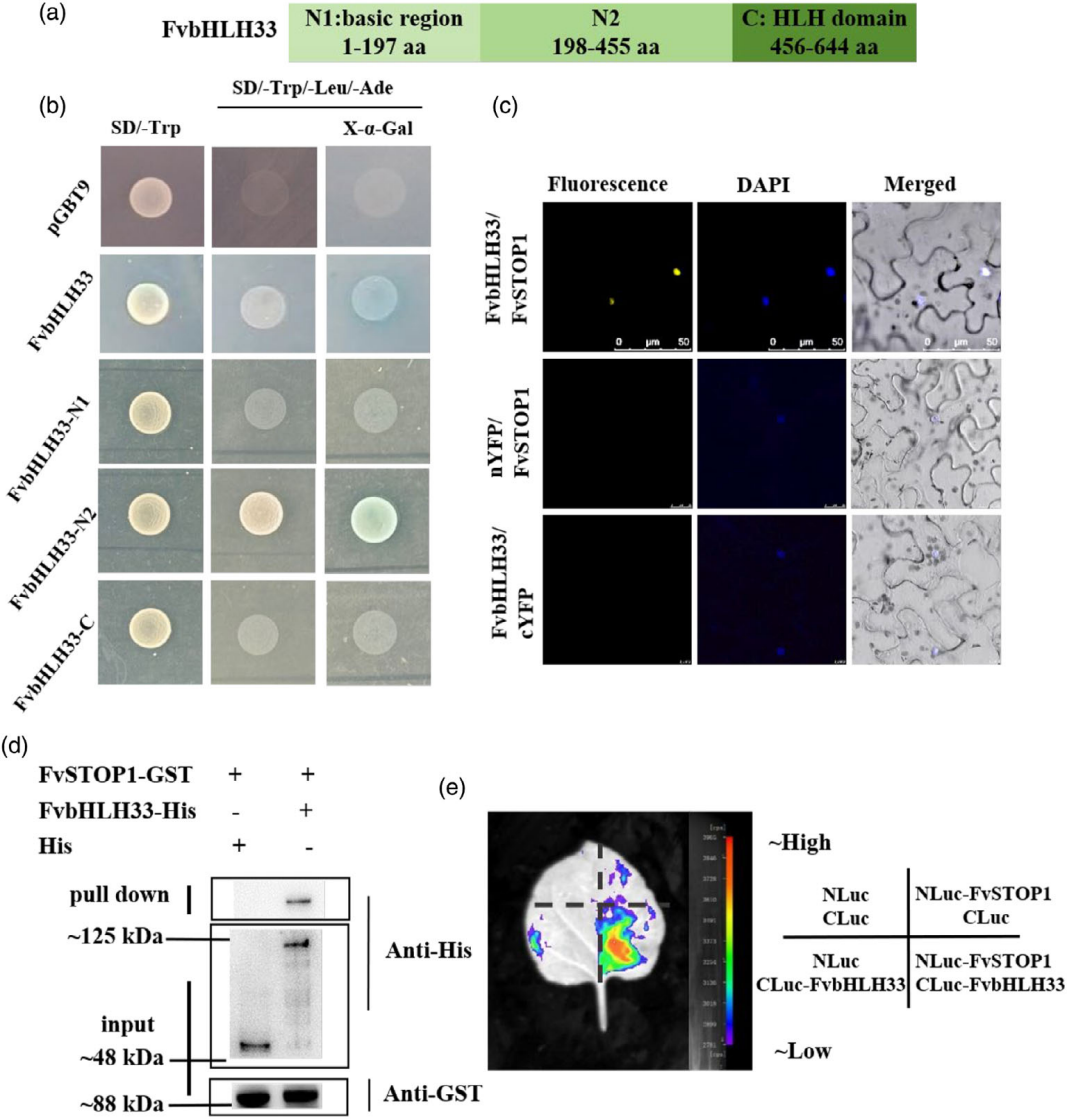
Analysis of the interaction between FvSTOP1 and FvbHLH33 *in vitro* and *in vivo*. (a) The segments of the FvbHLH33 protein, N1 (1‐197 aa), N2 (198–455 aa), C (456–644 aa). (b) Self‐activation activity of FvbHLH33. FvbHLH33‐pGBT9, FvbHLH33 (N1, 1–197 aa)‐pGBT9, FvbHLH33 (N2, 198–455 aa)‐pGBT9, FvbHLH33 (C, 456–644 aa)‐pGBT9 and pGBT9 (negative control) was transformed into Y2H gold cells and detected on agar plates (SD/−Trp and SD/−Trp/−Leu/−Ade/X‐α‐gal). (c) Bimolecular fluorescence complementation (BiFC) assay demonstrated the interaction between FvSTOP1 and FvbHLH33. The scale bar of FvbHLH33/ FvSTOP1 is 50 μm. The scale bar of cYFP/ FvSTOP1 is 25 μm. The scale bar of FvMYB1/nYFP is 10 μm. DAPI (4′,6‐Diamidino‐2′‐phenylindole) is a blue nuclear dye. (d) Pull‐down assays revealed the interaction between FvSTOP1 and FvbHLH33. The protein band emphasized by the anti‐His illustrated that FvSTOP1‐GST pulled down FvbHLH33‐His protein. (e) Luciferase complementation imaging assay (LCI) demonstrated that FvSTOP1 interacted with FvbHLH33.

FvSTOP1 had self‐activation activity in the yeast cells (Figure 1c). To further clarify whether FvbHLH33 had self‐activation activity and which regions of FvbHLH33 possessed self‐activation activity, we divided its full‐length coding sequence into three independent segments based on structural domains: N1 (1–197 aa; basic region), N2 (198–455 aa), and C (456–644 aa; HLH domain), and then performed a self‐activation assay (Figure 6a). Yeast cells carrying the FvbHLH33 N2 (198–455 aa) region grew well on selective media (SD/−Trp and SD/−Trp/−Leu/−Ade), whereas those harbouring the N1 (1–197 aa) or C (456–644 aa) regions showed no growth (Figure 6b). Next, we tested the interaction between FvSTOP1 and FvbHLH33 by BiFC, pull‐down, and LCI assays. We first co‐expressed FvbHLH33‐cYFP with nYFP‐FvSTOP1 in the leaves of tobacco. We found that the YFP signal was detected in the nucleus, but no fluorescence signal in the control (Figure 6c). We also conducted pull‐down assays to prove the interaction between FvSTOP1 and FvMYB1. Fusion protein FvbHLH33‐His and pCold‐TF (His) were incubated with FvSTOP1‐GST *in vitro*. Anti‐His and anti‐GST were used to perform the WB analysis, respectively. The results showed that FvbHLH33‐His could be pulled down, while pCold‐TF (His) could not (Figure 6d). In addition, the LCI assay further demonstrated that FvSTOP1 interacted with FvbHLH33. The co‐injection area of NLuc‐FvSTOP1 and FvbHLH33‐CLuc had an intense fluorescence signal, while no signals were observed in the control (NLuc‐FvSTOP1 and CLuc, NLuc and FvbHLH33‐CLuc, NLuc and CLuc) (Figure 6e). The above results suggested that FvSTOP1 not only interacted with FvMYB1 but also interacted with FvbHLH33.

### FvSTOP1 disturbs the formation of the FvMYB1–FvbHLH33 repression complex

**Figure 7:**
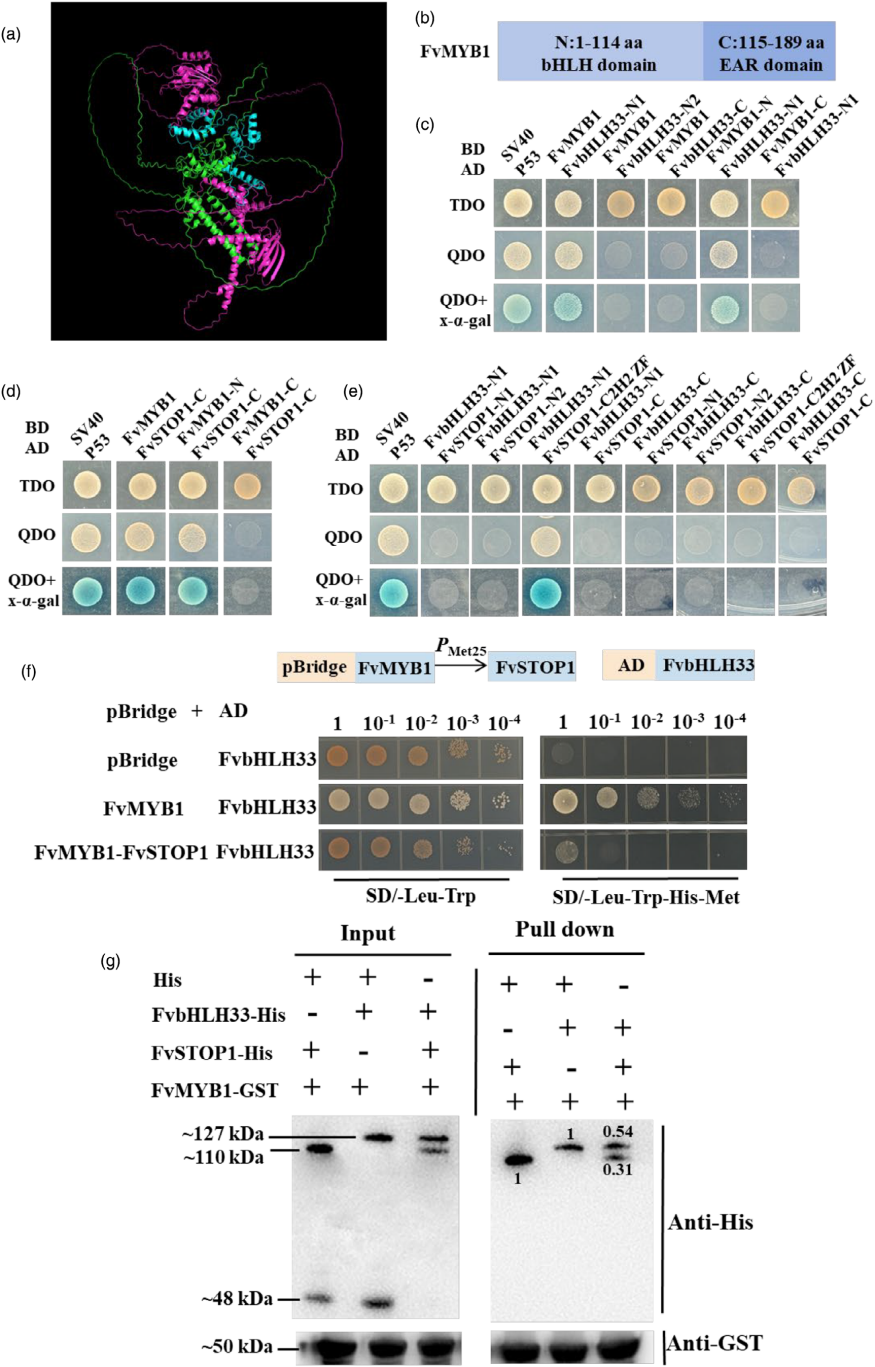
FvSTOP1 destroys the formation of the FvMYB1‐FvbHLH33 complex. (a) The AlphaFold3 software was used to predict the protein structure of the FvSTOP1–FvMYB1–FvbHLH33 ternary complex. The green protein structure represents FvSTOP1. The blue protein structure represents FvMYB1. The pink protein structure represents FvbHLH33. (b) The segments of the FvMYB1 protein are N1 (1–114 aa) and C (115–189 aa). (c) Yeast two‐hybrid (Y2H) assay detected the interaction between FvSTOP1 (C, 414–523 aa) and FvMYB1 (N, 1–114 aa). The positive control was the co‐transformation of SV40‐BD and P53‐AD. (d) Yeast two‐hybrid (Y2H) assay detected the interaction between FvMYB1 (N, 1–114 aa) and FvbHLH33 (N1, 1–197 aa). The positive control was the co‐transformation of SV40‐BD and P53‐AD. (e) Yeast two‐hybrid (Y2H) assay detected the interaction between FvbHLH33 (N1, 1–197 aa) and FvSTOP1 (C, 253–413 aa). The positive control was the co‐transformation of SV40‐BD and P53‐AD. (f) Yeast three‐hybrid (Y3H) assay was used to detect FvSTOP1 disturbing the formation of the FvMYB1–FvbHLH33 repression complex of anthocyanins. The growth of yeast harbouring FvMYB1–FvbHLH33 on SD/−Leu/−Trp agar plates and SD/−Met/−His/−Leu/−Trp agar plates was observed in the presence or absence of FvSTOP1. *P*_Met25_ is a methionine‐induced promoter. In the absence of methionine, FvSTOP1 was activated. (g) FvSTOP1 disturbed the formation of the FvMYB1–FvbHLH33 repression complex of anthocyanins by pull‐down assays. The interaction between FvMYB1‐FvbHLH33 in the presence or absence of FvSTOP1 was detected. ‘+’ and ‘−’ indicate the presence and absence, respectively. Pull‐down detection used anti‐His to incubate FvSTOP1‐His and FvbHLH33‐His.

FvSTOP1 interacts with FvMYB1 and FvbHLH33 to form the FvSTOP1–FvMYB1–FvbHLH33 ternary complex. How does this complex regulate anthocyanin accumulation? Based on the AlphaFold3 software (Abramson *et al*., 2024) prediction of the FvSTOP1–FvMYB1–FvbHLH33 ternary complex (Figure 7a), we proposed that FvSTOP1 interacts with FvMYB1 and FvbHLH33, thereby interfering with the formation of the FvMYB1–FvbHLH33 repression complex of anthocyanins and ultimately regulating the expression of anthocyanin structural genes.

To elucidate the interaction network among FvSTOP1, FvMYB1, and FvbHLH33, we conducted systematic domain analysis based on their structural characteristics. FvSTOP1 (523 aa) was divided into four functional segments: N1 (1–102 aa), N2 (103–252 aa), C2H2 ZF (253–413 aa, containing the zinc finger domain), and C‐terminal region (414–523 aa). Similarly, FvbHLH33 (644 aa) comprised three domains: N1 (1–197 aa, containing the basic region), N2 (198–455 aa), and C‐terminal region (456–644 aa, with HLH domain). FvMYB1 (189 aa) was partitioned into N‐terminal (1–114 aa, harbouring bHLH‐binding domain) and C‐terminal (115–189 aa, containing EAR domain) segments (Figure 7b). Through Y2H analysis, we identified three critical interaction pairs: (1) The N‐terminal bHLH‐binding containing region of FvMYB1 (1–114 aa) demonstrated dual binding capacity, interacting with both the basic region of FvbHLH33 N1 (1–197 aa) (Figure 7c) and the C‐terminal region of FvSTOP1 (414–523 aa) (Figure 7d). (2) Notably, the FvbHLH33 N1 domain (1–197 aa) also exhibited specific binding to the C2H2 zinc finger domain of FvSTOP1 (253–413 aa) (Figure 7e). This multi‐domain interaction pattern establishes that FvSTOP1 serves as a molecular bridge, connecting FvMYB1 through its C‐terminal domain while engaging FvbHLH33 via its zinc finger domain. Concurrently, FvMYB1 maintains direct interaction with FvbHLH33 through their respective N‐terminal domains. These domain‐specific interactions collectively support the formation of a ternary complex among the three proteins, providing structural basis for their functional coordination.

To detect whether FvSTOP1 disturbs the formation of the FvMYB1–FvbHLH33 repression complex of anthocyanins, we performed the yeast three‐hybrid (Y3H) assay. The Y3H system was used for investigating the influence of the third protein (FvSTOP1) on the interaction of two known interacting proteins (FvMYB1–FvbHLH33) by introducing the pBridge plasmid which simultaneously inserts two bait proteins (FvMYB1–FvSTOP1). FvSTOP1 as the third protein and FvMYB1 were inserted into pBridge to form the fusion vector FvMYB1–FvSTOP1–pBridge which was co‐transferred into Y2H gold cells with the fusion vector FvbHLH33‐AD. The pBridge and FvbHLH33‐AD vectors were used as the negative control. All strains grew normally on SD/−Leu/−Trp agar plates. FvMYB1 interacts with FvbHLH33; therefore, FvMYB1‐pBridge and FvbHLH33‐AD grew normally on SD/−Leu/−Trp/−His/−Met agar plates. When FvSTOP1 was added, the growth of FvMYB1–FvSTOP1–pBridge and FvbHLH33‐AD was greatly repressed on SD/−Leu/−Trp/−His/−Met agar plates compared with FvMYB1–pBridge and FvbHLH33‐AD, while the negative control could not grow. This indicated that FvSTOP1 destroyed the interaction between FvbHLH33 and FvMYB1 (Figure 7f). Moreover, we conducted a pull‐down assay to further verify whether FvSTOP1 affects the formation of the FvMYB1–FvbHLH33 repression complex of anthocyanins. When we incubated FvSTOP1‐His and FvMYB1‐GST, it suggested that FvSTOP1 interacted with FvMYB1. When we incubated FvbHLH33‐His and FvMYB1‐GST, it suggested that FvbHLH33 interacts with FvMYB1. When we incubated FvSTOP1‐His and FvbHLH33‐His together with FvMYB1‐GST, the binding strength of both with FvMYB1‐GST was significantly lower than when incubated alone with FvMYB1‐GST after pulldown. The binding strength was reduced by 0.31 and 0.54 times calculated by ImageJ software (Schroeder *et al*., 2021). These data indicated that FvSTOP1 hindered the interaction between FvMYB1 and FvbHLH33 (Figure 7g). Together, these results suggested that FvSTOP1 obstructed the formation of the FvMYB1‐FvbHLH33 repression complex of anthocyanins.

### FvSTOP1 disturbs the formation of the FvMYB1–FvbHLH33 repression complex to promote the expression of anthocyanin‐related genes

**Figure 8:**
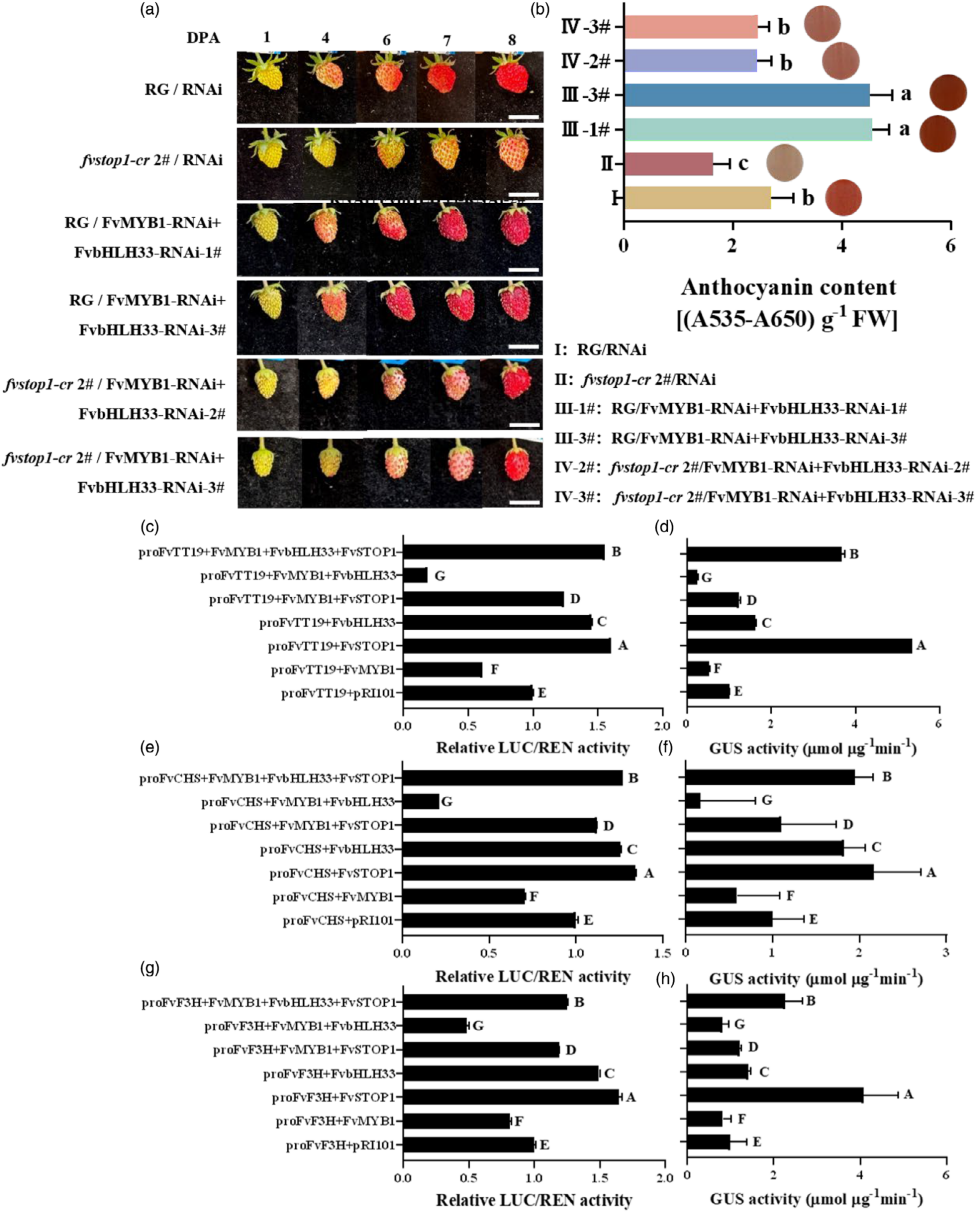
FvSTOP1 interferes with the formation of the FvMYB1–FvbHLH33 repression complex to repress the expression of the anthocyanin‐related gene. (a) Co‐injection of silencing *FvMYB1* and *FvbHLH33* (*FvMYB1*‐RNAi, *FvbHLH33*‐RNAi) into ‘Ruegen’ and knockout transgenic (*fvstop1*‐cr 2#) fruits (RG/*FvMYB1*‐RNAi *+ FvbHLH33*‐RNAi, *fvstop1*‐cr 2#/*FvMYB1*‐RNAi + *FvbHLH33*‐RNAi). The injected fruits were at the big green stage (18 days after pollination) and at least 15 fruits were selected for transient expression assays. Scale bar = 1 cm. (b) Anthocyanin contents in transient transformation fruits (*FvMYB1*‐RNAi and *FvbHLH33*‐RNAi injected into ‘Ruegen’ fruits and *fvstop1*‐cr 2# fruits). The circles represent the colour of the anthocyanin extract. FW is fresh weight. (c) Relative LUC/REN values of the activity of *FvTT19* promoter in tobacco with different combinations (*proFvTT19* + FvSTOP1 + FvMYB1 + FvbHLH33; *proFvTT19* + FvMYB1 + FvbHLH33; *proFvTT19* + FvSTOP1 + FvMYB1; *proFvTT19*+ FvbHLH33; *proFvTT19* + FvSTOP1; *proFvTT19* + FvMYB1; *proFvTT19* + pRI101). (d) GUS activity of *FvTT19* promoter in strawberry fruits with different combinations (same as the combinations in Figure 8c). (e) Relative LUC/REN values of the activity of *FvCHS* promoter in tobacco with different combinations (*proFvCHS* + FvSTOP1 + FvMYB1 + FvbHLH33; *proFvCHS* + FvMYB1 + FvbHLH33; *proFvCHS* + FvSTOP1 + FvMYB1; *proFvCHS* + FvbHLH33; *proFvCHS* + FvSTOP1; *proFvCHS* + FvMYB1; *proFvCHS* + pRI101). (f) GUS activity of *FvCHS* promoter in strawberry fruits with different combinations (same as the combinations in Figure 8e). (g) Relative LUC/REN values of the activity of *FvF3H* promoter in tobacco with different combinations (*proFvF3H* + FvSTOP1 + FvMYB1 + FvbHLH33; *proFvFvF3H* + FvMYB1 + FvbHLH33; *proFvF3H* + FvSTOP1 + FvMYB1; *proFvF3H* + FvbHLH33; *proFvF3H* + FvSTOP1; *proFvF3H* + FvMYB1; *proFvF3H* + pRI101). (h) GUS activity of *FvF3H* promoter in strawberry fruits with different combinations (same as the combinations in Figure 8g). Data are the mean ± SD of three biological replicates. Different letters represent significant differences compared with the control with Tukey's hoc test (*P* < 0.05).

To investigate whether FvSTOP1 interferes with the formation of the FvMYB1–FvbHLH33 repression complex to affect the expression of anthocyanin‐related genes in strawberry, we used transient expression assays in the fruits of ‘Ruegen’ and *fvstop1*‐*cr* 2# plants. The silencing *FvMYB1* and *FvbHLH33* bacterial suspension (*FvMYB1*‐RNAi and *FvbHLH33*‐RNAi) was injected into the fruits of ‘Ruegen’ and *fvstop1*‐*cr* 2# plants at the big green stage (18 days after pollination). After co‐injection of *FvMYB1*‐RNAi and *FvbHLH33*‐RNAi in ‘Ruegen’ fruits (RG/*FvMYB1*‐RNAi + *FvbHLH33*‐RNAi), the fruit colour turned redder, the anthocyanin content was 1.68 times higher than that of the control (Figure 8a,b) and the expression of anthocyanin‐related genes was significantly increased (Figure S9a). This indicated that the FvMYB1–FvbHLH33 complex negatively regulated anthocyanin biosynthesis. Compared with RG/*FvMYB1*‐RNAi + *FvbHLH33*‐RNAi, the fruit colour of co‐injection of *FvMYB1*‐RNAi and *FvbHLH33*‐RNAi in *fvstop1*‐*cr* 2# plant fruits (*fvstop1*‐*cr* 2#/*FvMYB1*‐RNAi + *FvbHLH33*‐RNAi) turned light red and the anthocyanin content was decreased by 50.0% (Figure 8a,b). The expression of anthocyanin‐related genes *FvCHS*, *FvANS*, *FvTT12*, and *FvTT19* in the fruit of *fvstop1*‐*cr* 2#/*FvMYB1*‐RNAi + *FvbHLH33*‐RNAi was remarkably decreased compared with the fruit (RG/*FvMYB1*‐RNAi + *FvbHLH33*‐RNAi) (Figure S9b). These results illustrate that FvSTOP1 interferes with the formation of the FvMYB1–FvbHLH33 repression complex to promote the expression of anthocyanin‐related genes.

To further examine how the FvSTOP1–FvMYB1–FvbHLH33 ternary complex regulates the expression of key genes that take part in anthocyanin accumulation, we ligated the promoters of *FvTT19*, *FvCHS*, and *FvF3H* to the LUC and GUS reporter genes and injected tobacco leaves and strawberry fruits, respectively. The LUC assay indicated that the activity of the *FvTT19* promoter was significantly inhibited by only injecting FvMYB1, while the *FvTT19* promoter activity was significantly activated by injecting FvSTOP1 and FvbHLH33 separately (Figure 8c). The *FvTT19* promoter activity in the co‐injection of FvMYB1 and FvbHLH33 was less than that of only the injection of FvMYB1, whereas the *FvTT19* promoter activity was greatly activated by co‐injection of FvSTOP1, FvMYB1, and FvbHLH33 (Figure 8c). These results indicated that FvSTOP1 significantly disturbed the inhibitory activity of FvMYB1 and FvbHLH33. The GUS assay in strawberry fruits was similar to the LUC assay in tobacco leaves (Figure 8d). In addition, the trends of anthocyanin biosynthetic genes *FvCHS* and *FvF3H* promoter activities regulated by different combinations of the FvSTOP1, FvMYB1, and FvbHLH33 were consistent with the results of *FvTT19* (Figure 8e–h). Taken together, FvSTOP1 disturbed the formation of the FvMYB1–FvbHLH33 repression complex of anthocyanins, thus promoting the anthocyanin accumulation by reducing the inhibition of the FvMYB1–FvbHLH33 complex on anthocyanin‐related genes in strawberry fruits.

## Discussion

### FvSTOP1 directly activates *FvTT19* to promote anthocyanin accumulation

Numerous studies have shown that the STOP1 transcription factor regulates plant adaptation to various environments (Sadhukhan *et al*., 2021). STOP1 regulates rhizospheric pH homeostasis, enhances nitrate uptake (Ye *et al*., 2021), enhances Al resistance (Zhou *et al*., 2023), responds to Pi deficiency (Wang *et al*., 2019), manages the trade‐off between salt and drought tolerance (Sadhukhan *et al*., 2019), and addresses low‐oxygen stress (Enomoto *et al*., 2019). However, whether STOP1 regulates anthocyanin accumulation remains unclear.

In this study, we found that *FvSTOP1* was highly expressed in the root and mature fruit of strawberries (Figure 1a). FvSTOP1 positively modulated anthocyanin accumulation in strawberries through stable overexpressing and knockout *FvSTOP1* plant analysis (Figure 3a,d). How does strawberry FvSTOP1 regulate anthocyanin accumulation? The expression of genes related to anthocyanin biosynthesis and transport in the fruits of overexpressing and knockout *FvSTOP1* plants was significantly increased and decreased, respectively (Figure 3b,e). Surprisingly, Y1H assay analysis found that FvSTOP1 only bound to the promoter of *FvTT19*, while FvSTOP1 could not bind to *FvCHS*, *FvF3H*, *FvDFR*, *FvANS*, and *FvTT12*. The promoters of *FvCHS*, *FvF3H*, *FvDFR*, *FvANS*, and *FvTT12* genes contained the binding motif GGN(T/g/a/C)V(C/A/g)S(C/G)T of STOP1 (Liu *et al*., 2016; Figure S6). The possible reason for this phenomenon is that the binding motif of STOP1 reported in previous literature may respond to environmental stresses, while the binding motifs of secondary metabolism such as anthocyanins may differ with environmental stresses. Therefore, we transiently silenced *FvTT19* in the fruit of overexpressing *FvSTOP1* plants and found that anthocyanin accumulation in fruits was greatly reduced compared with the control. These results demonstrated that *FvTT19* was the direct target of FvSTOP1.

Transient functional analysis of *FvTT19* showed that the colour of overexpressing and silencing *FvTT19* fruits turned dark red and light red, respectively (Figure S8a). *RAP* obtained by N‐ethyl‐N‐nitrosourea (ENU) mutagenized in woodland strawberry encodes a *GST* gene that promotes anthocyanin accumulation (Luo *et al*., 2018). Interestingly, *RAP* also positively regulated anthocyanin accumulation and the sequence of *FvTT19* in this study was consistent with *RAP* (Figure S10). *Arabidopsis* TT19 is a GST and TT19 is not only located in the cytoplasm and tonoplast but also in the nuclei, which transfers anthocyanin from the ER to the tonoplast (Sun *et al*., 2012). *Arabidopsis* TT19 mainly binds cyanidin (Cya) on the ER and binds to cyanidin‐3‐*O*‐glycoside (Cy3G) in the cytoplasm (Sun *et al*., 2012). In the cytosol, the flavonoids bond with TT19 and are modified by acylation and glycosylation. Ultimately, the acylated anthocyanins are liberated from TT19, absorbed by a transporter situated on the tonoplast, and eventually sequestered into the vacuole in *Arabidopsis* (Springob *et al*., 2003; Sun *et al*., 2012; Yonekura‐Sakakibara *et al*., 2009). Here, FvSTOP1 was directly bound to *FvTT19* and promoted its expression, which increased the content of anthocyanin in fruits. FvTT19 might enhance anthocyanin transport by increasing the binding with Cya and Cy3G, thus leading to anthocyanin accumulation.

### FvSTOP1 interferes with the formation of the FvMYB1–FvbHLH33 repression complex to regulate anthocyanin accumulation

The MBW activation ternary complex plays a vital role in the regulation of secondary metabolites such as flavonoids (Lin‐Wang *et al*., 2014; Schaart *et al*., 2012). Several transcription factors regulate anthocyanin biosynthesis by interacting with the MBW activation complex (Chen *et al*., 2019). Among them, many anthocyanin repressors are reported to interact with the components of the MBW activation complex to inhibit anthocyanin biosynthesis by interfering with the formation of the MBW complex (Chen *et al*., 2019). Peach R3‐MYB repressor PpMYB18 regulates the biosynthesis of anthocyanins and proanthocyanidins by competing with MYB activators PpMYBPA1 and PpMYB10.1 to bind to PpbHLH3 or PpbHLH33 (Zhou *et al*., 2018). Pear R2R3‐MYB repressor PpMYB140 competes with anthocyanin‐activated MYB to bind bHLH and hamper the formation of the MBW complex, thereby repressing anthocyanin biosynthesis (Ni *et al*., 2020). Similar to PpMYB18 and PpMYB140, many MYB repressors that inhibit anthocyanin biosynthesis by competitively binding bHLH with MYB activator of the MBW complex have been identified, including R2R3‐MYB SlMYB7 from tomato (Zhang *et al*., 2023a), R2R3‐type PhMYB27 from *Petunia* (Albert *et al*., 2014), R3‐MYB FhMYBx from *Freesia* (Li *et al*., 2020), R3‐MYB BnCPC from rapeseed (Xie *et al*., 2022). In addition, some bHLH repressors of anthocyanins competitively bind to another bHLH with R2R3‐MYB activators have also been reported. Apple anthocyanin repressor MdbHLH162 competitively binds to MdbHLH3/MdbHLH33 with anthocyanin activator MdMYB1 and destroys the formation of the MdMYB1‐MdbHLH3/33 activation complex, thus reducing anthocyanin biosynthesis (An *et al*., 2024).

Besides MYB and bHLH transcription factors, other types of transcription factors that repress anthocyanin biosynthesis by disturbing the formation of activation complexes have been identified. Such as pear B‐box protein PpBBX21 negatively regulates anthocyanin accumulation. Pear PpBBX18 interacts with PpHY5 to activate the expression of PpMYB10 and promote anthocyanin biosynthesis. However, when PpBBX21 interacts with PpBBX18 and PpHY5, it destabilizes the PpBBX18‐PpHY5 complex and represses anthocyanin biosynthesis (Bai *et al*., 2019). Pear PyERF4.1/PyERF4.2 interacts with PyERF3 to destabilize the PyERF3–PyMYB114–PybHLH3 activation complex, decreasing the expression of the anthocyanin biosynthetic gene *PyANS* and anthocyanin biosynthesis (Sun *et al*., 2023). *Arabidopsis* SBP‐box protein AtSPL9 negatively regulates anthocyanin accumulation. AtSPL9 competitively binds to AtPAP1 with AtTT8 and interferes with activation complex AtPAP1–AtTT8–AtTTG1 formation to inhibit anthocyanin biosynthesis (Gou *et al*., 2011).

The above information indicates that transcriptional repressors play an inhibitory role in anthocyanin biosynthesis by disrupting the stability of the MBW or other activation complexes through protein–protein interaction (LaFountain and Yuan, 2021). However, the studies on transcriptional activators that interfered with the formation of MBW and other repression complexes to promote anthocyanin biosynthesis were fewer. Banana proanthocyanidin R2R3 MYB activator MaMYBPA competitively binds to MaMYC with repressor MaMYBPR and disrupts the formation of the repression complex MaMYBPR–MaMYC–MaTTG1, thereby reducing the inhibition of repression complex MaMYBPR–MaMYC–MaTTG1 to promote proanthocyanidins biosynthesis (Rajput *et al*., 2022). *Arabidopsis* DELLA protein AtRGA competitively binds to anthocyanin repressor AtMYBL2 with AtEGL3 and hinders the interaction between AtMYBL2 and activation complex AtPAP1–AtEGL3–AtTTG1, thus reducing the inhibition of repression complex AtMYBL2–AtPAP1–AtEGL3–AtTTG1 to promote the expression of *AtDFR* and anthocyanin biosynthesis (Dubos *et al*., 2008; Li *et al*., 2020). In this study, we found that FvSTOP1 promoted anthocyanin accumulation by disturbing the formation of the FvMYB1–FvbHLH33 repression complex to weaken the inhibition of the FvMYB1–FvbHLH33 on the expression of anthocyanin‐related genes.

**Figure 9:**
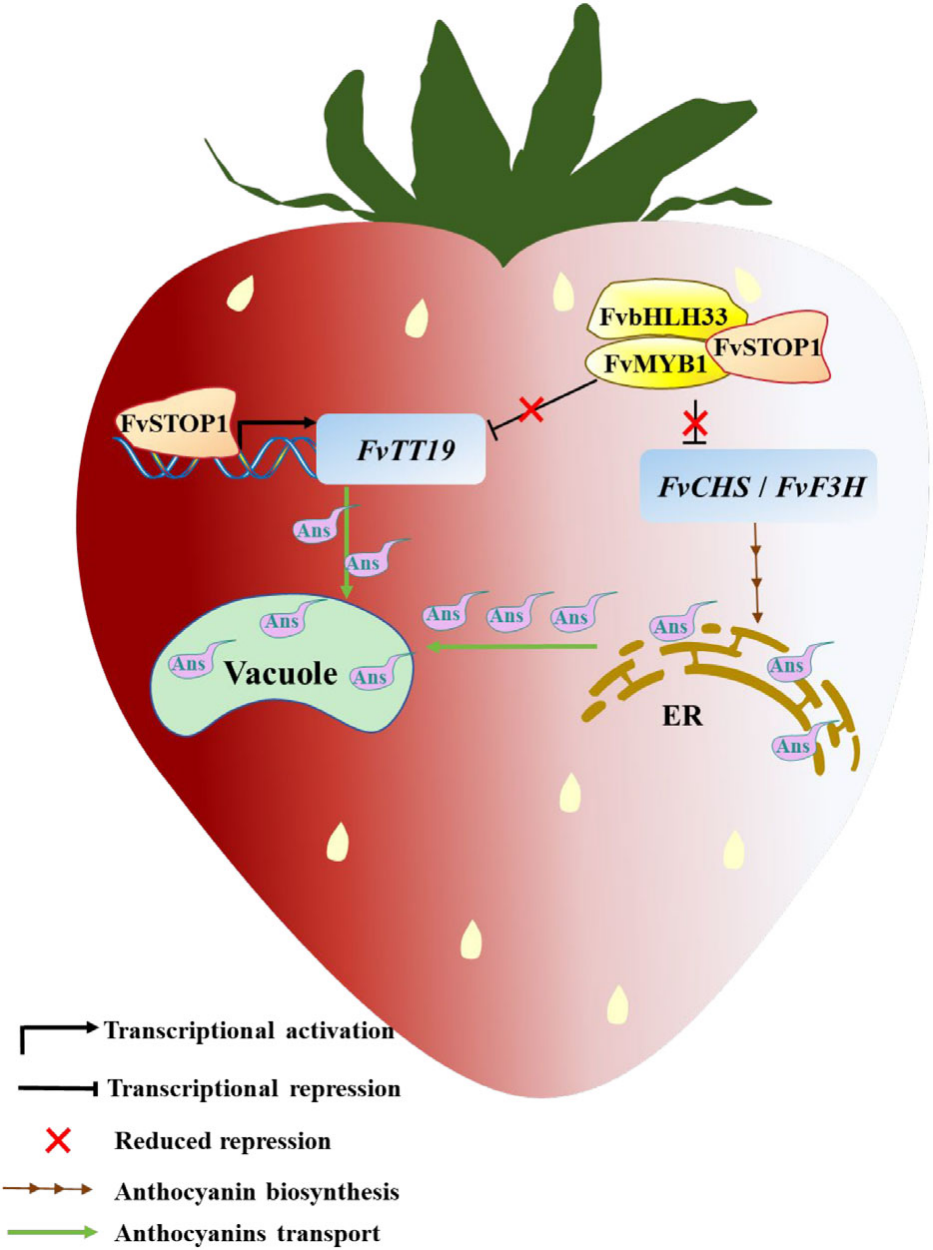
A working model of FvSTOP1 promoting anthocyanin accumulation in strawberry fruits. On the one hand, FvSTOP1 promotes anthocyanin transport and accumulation by directly binding to the *FvTT19* promoter. On the other hand, FvSTOP1 and FvMYB1–FvbHLH33 form a ternary complex. FvSTOP1 interferes with the formation of the FvMYB1–FvbHLH33 repression complex of anthocyanins to reduce the repression of anthocyanin‐related gene expression, thereby promoting anthocyanin accumulation. The orange block represents FvSTOP1. The yellow block represents the FvMYB1–FvbHLH33 complex. The blue block represents anthocyanin‐related genes (*FvTT19*, *FvCHS*, *FvF3H*). Ans represents anthocyanin. The green block represents a vacuole. The brown area represents the endoplasmic reticulum (ER).

In summary, strawberry FvSTOP1 promoted anthocyanin accumulation by inducing the expression of *FvTT19* and interfering with the formation of the FvMYB1–FvbHLH33 repression complex of anthocyanins (Figure 9). This study discovered the new function of STOP1 in plant species, and FvSTOP1 will be useful for improving the colour and quality of strawberry.

## Materials and methods

### Plant materials and growth conditions

‘Ruegen’ (*F. vesca*), the diploid woodland strawberry was grown in the greenhouse of Shenyang Agricultural University, China.

Overexpressing and knockout *FvSTOP1* transgenic strawberry plants and WT (‘Ruegen’) strawberry plants were identified in culture medium (0.443 g/L MS, 30 g/L sucrose, 0.4 mg/L 2,4‐dichlorophenoxyacetic acid, 0.1 mg/L Indole‐3‐butyric acid, pH = 5.4) and then transferred in soil substrate (peat: vermiculite: perlite = 2:1:1) cultivating at 16 h in light and 8 h in dark condition in lighting incubator. The photosynthetic photon flux density (PPFD) in the lighting incubator was maintained at 269.8 μmol·m^−2^·s^−1^, with spectral ratios of red (630 nm), green (550 nm), and blue (450 nm) light at 39.4%, 33.1%, and 33.1%, respectively (Yao *et al*., 2025). And 28 days later, the strawberry plants were moved to the greenhouse of Shenyang Agricultural University. In the greenhouse, PPFD measurements showed diurnal variations: 251.4 μmol·m^−2^·s^−1^ with red/green/blue spectral ratios of 45.8%/30.4%/23.8% at 08:00, increasing to 640.0 μmol·m^−2^·s^−1^ with ratios of 44.8%/31.1%/24.1% at 13:00.

Tobacco (*Nicotiana benthamiana*) plants were grown in soil substrate (peat: vermiculite = 1:1) at 23 °C (18 h/6 h, light/dark) for 4 weeks.

### Phylogenetic analysis and sequence analysis

STOP1 protein sequences from various crops were obtained from the NCBI database (https://www.ncbi.nlm.nih.gov/). ClustalW (http://www.clustal.org/) was used to align the phylogenetic analysis. The phylogenetic tree was performed with MEGA 6.0 (http://www. megasoftware.net/) (Tamura *et al*., 2013) based on the neighbour‐joining method. DNAMAN software was used for comparing amino acid sequences of STOP1 from strawberry and other plant species (default parameter, Lynnon Biosoft, Quebec, Canada).

### RT‐qPCR analysis

Different organs from ‘Ruegen’ including roots, petiole, leaf, shoot apex, flower, and four development stages of fruits (small green, white, turning, and red; 10, 17, 24, and 30 days after pollination, respectively) were frozen in liquid nitrogen and stored at −80 °C for quantitative RT–qPCR analysis. RT‐qPCR analysis used the 7500 Real‐Time PCR System (Applied Biosystems, Foster City, California, USA). RT‐qPCR assay was based on the method of Bian *et al*. (2022). All data were normalized first with the level of the 26S internal transcript control and then with the expression of controls (Zhang *et al*., 2023b). Biological and examined replicates with triplicate in each sample.

### Subcellular localization

RT‐PCR was used to amplify *FvSTOP1* from the leaf of the ‘Ruegen’. Constructing the fusion vector *p35S*::FvSTOP1‐GFP used restriction enzymes *Sal*I and *BamH*I. *Agrobacterium tumefaciens* cells with GFP and FvSTOP1‐GFP were infiltrated in 4‐week‐old tobaccos. About 2 days later, the confocal fluorescence microscopy was used to scan the fluorescence signal of GFP (Leica DMi8 A, Wetzlar, Germany).

### Transient and stable transformation of woodland strawberry

Constructing the overexpression vector of *FvSTOP1* (*p35S*::*FvSTOP1*‐3Flag) utilized restriction enzymes *Nde*I and *EcoR*I. The primer sets are shown in Table S1. Transformed pRI101‐AN (control) and *p35S*::*FvSTOP1* plasmids into the *Agrobacterium tumefaciens* strain GV3101. The CRISPR/Cas9 knockout vector of *FvSTOP1* (*FvSTOP1*‐cr) construction was according to the method reported previously by Zhu *et al*. (2018). Designed the sgRNA1 and sgRNA2 targeting the *FvSTOP1* by the website CRISPR‐P 2.0 (http://crispr.hzau.edu.cn/CRISPR2/). The sgRNA1/2 was constructed and inserted into the entry vector with the AtU3d promoter by *Bsa*I. The two expression cassettes with sgRNA1 (Target 1: 5′‐GAAACTCAGCCAGCCATCTG‐3′) and sgRNA2 (Target 2: 5′‐GCTCAATTGATCTCTACGGC‐3′) were then incorporated into the pYLCRISPR/Cas9 Pubi‐N binary plasmid used Golden Gate ligation as reported by Ma *et al*. (2015).

The transient transformation assay in fruits was carried out using the method reported by Zhang *et al*. (2024). *Agrobacterium*‐mediated transient transformation of strawberry fruit is a fast and universal method for studying the functions of genes during fruit ripening. The strawberry fruits were injected at the big green fruit stage (18 days after pollination). According to our experiments, the transient transformation efficiency was about 60%.

The stable transformation of woodland strawberry was carried out by *Agrobacterium tumefaciens* strain GV3101 as described by Li *et al*. (2017b). Positive transgenic plants of overexpressing plants were grown on the selected medium (2.47 g/L 1/2 MS medium + 30 g/L sucrose + 30 mg/L kanamycin, pH 5.6). Positive transgenic plants of knockout plants were identified by Sanger sequencing. The genotypes of the gene‐edited plants were analysed based on the online website (http://skl.scau.edu.cn/dsdecode/).

### Self‐activation activity

*FvSTOP1* was amplified and ligated to the pGBT9 vector using restriction enzymes *EcoR*I and *Pst*I, while the N1‐terminal of the FvSTOP1 (N1, 1–102 aa), N2‐terminal of the FvSTOP1 (N2, 103–252 aa), C2H2 ZF‐terminal of the FvSTOP1 (C2H2 ZF, 253–413 aa) and C‐terminal of the FvSTOP1 (C, 414–523 aa) coding regions were inserted into the DNA‐binding domain vector pGBT9. FvSTOP1‐pGBT9, FvSTOP1(N1)‐pGBT9, FvSTOP1(N2)‐pGBT9, FvSTOP1(C2H2 ZF)‐pGBT9, FvSTOP1(C)‐pGBT9, and pGBT9 (negative control) were transformed into Y2H gold cells and cultured on SD/−Trp agar plates.

*FvbHLH33* was amplified and ligated to the pGBT9 vector using restriction enzymes *BamH*I and *Sal*I, while the FvbHLH33 (N1, 1–197 aa, including the basic region), FvbHLH33 (N2, 198–455 aa), and FvbHLH33 (C, 456–644 aa, including the HLH domain) coding regions were inserted into the DNA‐binding domain vector pGBT9. FvbHLH33‐pGBT9, FvbHLH33 (1–197 aa)‐pGBT9, FvbHLH33 (198–455 aa)‐pGBT9, FvbHLH33 (456–644 aa)‐pGBT9, and pGBT9 (negative control) were transformed into Y2H gold cells and cultured on SD/−Trp agar plates.

3 days later, positive single colonies were selected and cultured on SD/−Trp and SD/−Trp/−Leu/−Ade/X‐α‐Gal agar plates at 30 °C and observed the yeast cells 3 days later.

### Yeast one‐hybrid assay

The *FvSTOP*1 coding region was ligated to the pGADT7 vector using restriction enzymes *EcoR*I and *Pst*I. The promoter region of *FvCHS*, *FvF3H*, *FvDFR*, *FvANS*, *FvTT12*, and *FvTT19* was segmented and ligated to the pAbAi vector using restriction enzymes *Sac*I and *Sal*I, *Hind*III and *Sac*I, respectively. The fragments were as follows: *proFvCHS* (*proFvCHS1‐1*: ‐1790‐‐1548 bp, *proFvCHS1‐2*: ‐857‐‐568 bp, *proFvCHS2*: ‐1570‐‐836 bp, *proFvCHS3*: ‐588‐‐1 bp), *proFvF3H* (*proFvF3H‐1*: ‐2001‐‐1032 bp, *proFvF3H‐2*: ‐1091‐‐608 bp *proFvF3H‐3*: ‐646‐‐1 bp), *proFvDFR* (*proFvDFR‐1*: ‐2253‐‐1638 bp, *proFvDFR‐2*: ‐1698‐‐1042 bp, *proFvDFR‐3*: ‐1113‐‐463 bp, *proFvDFR‐4*: ‐552‐‐1 bp), *proFvANS* (‐2001‐‐1 bp), *proFvTT12* (*proFvTT12‐2*: ‐1056‐‐481 bp, *proFvTT12‐3*: ‐552‐‐1 bp), *proFvTT19* (*proFvTT19‐1*: ‐1455‐‐913 bp, *proFvTT19‐2*: ‐975‐‐479 bp, *proFvTT19‐3*: ‐534‐‐1 bp). pGADT7 was used as the negative control. The plasmids harbouring promoters‐pAbAi and FvSTOP1‐AD were transferred into the Y1H gold cells and cultured on SD/−Ura agar plates. About 2 days later, positive single colonies were selected and transferred to SD/−Leu agar plates without AbA and SD/−Leu/+AbA (25–500 μg/L) agar plates, and the growth state of yeast was observed in a 30 °C incubator 3 days later.

### Yeast two‐hybrid assay

The *FvSTOP1* was ligated to the pGADT7 vector using restriction enzymes *BamH*I and *SalI*. The *FvMYB1* was ligated to the pGBT9 vector using restriction enzymes *EcoR*I and *BamH*I, respectively. FvSTOP1‐pGADT7, FvSTOP1 (N1, 1–102 aa)‐pGADT7, FvSTOP1 (N2, 103–252 aa)‐pGADT7, FvSTOP1 (C2H2 ZF, 253–413 aa)‐pGADT7, FvSTOP1 (C, 414–523 aa)‐pGADT7, and FvMYB1‐pGBT9, FvMYB1 (N, 1–114 aa)‐pGBT9, FvMYB1 (C, 115–189 aa)‐pGBT9 were transferred into the Y2H gold cells, respectively.

The *FvbHLH33* was ligated to the pGADT7 vector using restriction enzymes *EcoR*I and *BamH*I. FvbHLH33 (N1, 1–197 aa)‐pGADT7, FvbHLH33 (N2, 198–455 aa)‐pGADT7, FvbHLH33 (C, 456–644 aa)‐pGADT7, and FvMYB1‐pGBT9, FvMYB1 (N, 1–114 aa)‐pGBT9, FvMYB1 (C, 115–189 aa)‐pGBT9 were transferred into the Y2H gold cells, respectively. FvbHLH33 (N1, 1–197 aa)‐pGADT7, FvbHLH33 (N2, 198–455 aa)‐pGADT7, FvbHLH33 (C, 456–644 aa)‐pGADT7, and FvSTOP1‐pGBT9, FvSTOP1 (N1, 1–102 aa)‐pGBT9, FvSTOP1 (C2H2 ZF, 253–413 aa)‐pGBT9, FvSTOP1 (C, 414–523 aa)‐pGBT9 were transferred into the Y2H gold cells, respectively.

These cells were cultured on a TDO agar plate (SD/−Trp/−Leu). And 2 days later, positive single colonies were selected and cultured on QDO agar plates (SD/−Trp/−Leu/−His/−Ade/X‐α‐gal) at 30 °C, and observed the yeast cells 3 days later.

### Yeast three‐hybrid assay

The assay method was conducted based on the pBridge manufacturer's manual (Clontech, Takara, Japan). *FvMYB1* was inserted into the pBridge vector to generate pBridge‐FvMYB1 using restriction enzyme *EcoR*I and *BamH*I. *FvSTOP1* was ligated to the fusion vector pBridge‐FvMYB1 to generate FvSTOP1‐FvMYB1 using restriction enzymes *Not*I and *Bgl*II. Then co‐transferred AD‐FvbHLH33 and FvSTOP1–FvMYB1–pBridge into the Y2H gold cells. The strains of yeast were grown on SD/−Trp/−Leu agar plates for 2 days. The cells with AD‐FvbHLH33 and FvMYB1‐pBridge, AD‐FvbHLH33 and FvSTOP1–FvMYB1–pBridge, AD‐FvbHLH33 and pBridge were transferred to SD/−Trp/−Leu/−His/−Met agar plates for 3 days to be observed.

### Measurement of total anthocyanins

The measurement method followed that described by Castro *et al*. (2018). Mature strawberry fruits (WT, FvSTOP1‐OE, *fvstop1‐cr*) at the same developmental stage were harvested 25 days after flowering, immediately frozen in liquid nitrogen, and ground to a fine powder. Approximately, 0.25 g of freeze‐dried powder was weighed and mixed with 5 mL of extraction solution (H_2_O: propanol: hydrochloric acid = 81:18:1, v/v/v). The mixture was boiled at 98 °C for 3 min in a preheated water bath, then incubated overnight in the dark. The following day, the solution was centrifuged at 12 000 rpm for 3 min, and the supernatant was transferred to a cuvette for spectrophotometric analysis (Hoefer Vision, SP‐2001). Absorbance values were measured at 535 nm (A_535_) and 650 nm (A_650_), with the extraction solution serving as the blank (zero‐reference). The content of anthocyanin was calculated according to the following formula: Anthocyanin = (A_535_–A_650_)/0.25 g. A_535_–A_650_ is the absorbance at the indicated wavelengths. The unit of total anthocyanin content is (A_535_–A_650_)/FW. Three independent repeated tests were performed on all samples.

### Flavonoid metabolomics analysis

Strawberry fruit powder (100 mg) was resuspended in pre‐cooled 80% methanol. The samples were incubated on ice for 5 min, then centrifuged at 15 000 × ***g*** for 20 min at 4 °C. The supernatant was collected and diluted with LC–MS‐grade water to a final methanol concentration of 53%. The diluted solution was centrifuged again under the same conditions (15 000 × ***g***, 4 °C, 20 min), and the resulting supernatant was injected into the LC–MS/MS system for analysis.

LC–MS/MS analysis was performed using an ExionLC™ AD system (SCIEX) coupled to a QTRAP® 6500+ mass spectrometer (SCIEX; Novogene, Beijing, China). Separation was achieved using a Hypersil Gold column with an XSelect HSS T3 stationary phase (2.1 × 150 mm, 2.5 μm). The mobile phase consisted of eluent A (0.1% formic acid in water) and eluent B (0.1% formic acid in acetonitrile). A 20‐min linear gradient was applied at a flow rate of 0.4 mL/min as follows: 2% B, 2 min; 2%–100% B, 15.0 min; 100% B, 17.0 min; 100%–2% B, 17.1 min; 2% B, 20 min. The detection of the experimental samples using multiple reaction monitoring (MRM) was based on Novogene in‐house database.

### GUS reporter assay

Overexpression of *FvSTOP1* (*p35S*::*FvSTOP1*) and pRI101‐AN acted as an effector. The promoters of *FvCHS*, *FvF3H*, and *FvTT19* were inserted into the pRI201‐AN‐GUS vector and were used as a reporter. The restriction enzymes *Pst*I and *Xba*I were used in constructing the fusion plasmids. We detected the GUS fluorescence activity following the method described by Li *et al*. (2017a).

### Dual‐luciferase reporter assay

Some 2000 bp of the promoter region of *FvCHS*, *FvF3H*, and *FvTT19* were selected and ligated to the pGreenII0800‐LUC vector. *proFvCHS*‐LUC, *proFvF3H*‐LUC, and *proFvTT19*‐LUC were used as reporter plasmids. The effector plasmids were overexpression of *FvSTOP1*, *FvMYB1*, and *FvbHLH33* (*p35S*::*FvSTOP1*, *p35S*::*FvMYB1*, *p35S*::*FvbHLH33*). The optical density at 600 nm (OD_600_) of *Agrobacterium* in different combinations was adjusted to 0.8 with bacterial suspension. The *Agrobacterium* mixture was placed in a 28 °C incubator for 2 h and then injected into 4‐week‐old tobacco leaves. The tobacco was injected and cultured for 3 days (2 days in dark and 1 day in light). The living fluorescence imager (Lb985, Berthold, Germany) was used to observe the luciferase signal. The LUC/REN ratio was calculated according to a dual luciferase reporter gene assay kit (Beyotime, Shanghai, China). Each value represents six biological replicates.

### Luciferase complementation imaging assays

*FvSTOP1* was ligated into the pCAMBIA1300‐NLuc vector using restriction enzymes *Kpn*1 and *Sal*l. *FvMYB1* and *FvbHLH33* were inserted into the pCAMBIA1300‐CLuc vector using restriction enzymes *Kpn*I and *BamH*I. The *Agrobacterium* mixture (OD_600_ = 0.8) was injected into the 4‐week‐old tobacco leaves. About 2 days later, a living fluorescence imager (Lb985, Berthold, Germany) was used to detect the luciferase signalling.

### Pull‐down assays

*FvMYB1* and *FvbHLH33* were ligated into the pCold‐TF vector. *FvSTOP1* was introduced into the pGEX‐4T‐1 vector. The FvSTOP1‐GST and FvMYB1‐His were transformed in BL21 (DE3) cells. We purified His‐fused proteins (His, FvMYB1‐His, FvbHLH33‐His) using a commercial kit (CWbio, Beijing, China). WB test was performed with anti‐GST and anti‐His antibodies (Abmart, Shanghai, China).

### BiFC assays

*FvSTOP1* was ligated into the pSPYNE vector, and *FvMYB1* and *FvbHLH33* were ligated into the pSPYNCE vector using restriction enzymes *BamH*I and *Kpn*I. The *Agrobacterium* mixture (OD_600_ = 0.8) was injected into the 4‐week‐old tobacco leaves. And 2 days later, the YFP fluorescence signal from FvSTOP1‐pSPYNE + FvMYB1‐pSPYNCE and FvSTOP1‐pSPYNE + FvbHLH33‐pSPYNCE was observed using confocal fluorescence microscopy (Leica DMi8 A, Wetzlar, Germany). DAPI (DNA dye 4′,6‐diamidino‐2'‐phenylindole) was used to label the cells to visualize the nucleus described by Wang *et al*. (2023).

### Western blot assays

The total protein of the mature fruits (30 days after pollination) of overexpressing and knockout *FvSTOP1* and WT was extracted by lysate (RIPA 100 μL + PMSF 100 mM, Beyotime, Shanghai, China). The extracted proteins were boiled. Then the boiled proteins were subjected to SDS‐PAGE using a running buffer (Servicebio, Wuhan, China) and transferred to a PVDF membrane using transfer buffer (Tris 3.03 g/L, glycine 14.42 g/L, methanol 100 mL). The PVDF membranes were first incubated in 5% sealing fluid for 2 h and washed with TBST five times (5 min each time). Then anti‐MYB1 and the internal reference Actin antibody were incubated with a 2.5% sealed solution. The membrane was incubated for 2 h with a 0.5% sealing solution containing horseradish peroxidase‐conjugated affineur goat anti‐rabbit IgG (anti‐HRP) antibodies at room temperature. After being washed with TBST buffer (Servicebio, Wuhan, China), blots were visualized by Tanon‐5200 Luminescent imaging (Tanon, Shanghai, China).

### Statistical analysis

Student's *t*‐test calculated statistical analysis (*P < 0.05, **P < 0.01, ***P < 0.001) and one‐way analysis of variance (ANOVA) combined with Tukey's hoc test (*P* < 0.05) by DPS 9.01 software. Data analysis was visualized and utilized for GraphPad Prism 7 (San Diego, California, USA).

## Accession numbers

Sequence data in this study can be found in the NCBI (https://www.ncbi.nlm.nih.gov/) databases: ZjSTOP1 (XP_015886371.3), TcSTOP1 (XP_007020278.1), CiSTOP1 (XP_042963050.1), CmSTOP1 (XP_008461471.1), DzSTOP1 (XP_022718212.1), CuSTOP1 (GAY39642.1), MrSTOP1 (KAB1220678.1), CsSTOP1 (XP_004139705.1), McSTOP1 (XP_022135444.1), AtSTOP1 (NP_174697.1), FvSTOP1 (XP_004290434.1), MdSTOP1 (XP_008365851.3), GmSTOP (XP_003556206.1), SbSTOP1 (XP_021311660.1), OsART1 (ATU81902.1), MsSTOP1 (XP_024641404.1), TaSTOP1 (XP_044351087.1), RcSTOP1 (XP_024163281.1), PpSTOP1 (XP_020425968.1), PaSTOP1 (KAH0976512.1), VvSTOP1 (XP_019072266.1), PmSTOP1 (XP_008237278.1), MbSTOP1 (TQE10061.1), LfSTOP1 (KAK9271294.1), CaSTOP1 (XP_059455480.1), CfSTOP1 (KAE8100290.1), RrSTOP1 (XP_062019448.1), EgSTOP1 (XP_010062013.1), DlSTOP1 (XP_052202179.1), ToSTOP1 (POO00227.1), JcSTOP1 (XP_012081095.1), QsSTOP1 (XP_023905586.1), MaSTOP1 (KAJ4701162.1), FvCHS (XM_004306495.2), FvF3H (XM_004287766.2), FvDFR (KC_894050.1), FvANS (XM_004298672.2), FvTT12 (NC_020496.1), and FvTT19 (XM_004288530.2).

## Author contributions

Ruiqing Bian conducted experiments, analysed data, and wrote the manuscript. Jinxiang Yao and Yuxin Nie conducted experiments. Yuying Zhang and Zhengjia Wu collected plant materials. Junxiang Zhang and Zhihong Zhang designed the experiments and modified the manuscript. All authors in this study read and approved the manuscript.

## Funding

This work was supported by the National Natural Science Foundation of China (32130092, 32272681), a sub‐project of Liaoning Province Germplasm Innovation Grain Storage and Technology Special Program (2023JH1/10200003), and Shenyang Young and Middle‐aged Science and Technology Innovation Talents Support Plan (RC220306).

## Conflict of interest

The authors declare no competing interests.

## Supporting information


**File S1** The coding sequences of *FvSTOP1* (XP_004290434.1), *FvMYB1* (XM_004299494.2), *FvbHLH33* (*XM_004308329.2*).

**Table S1** Primer sets were used in this study.

**Figure S1** Phylogenetic and structural analyses of FvSTOP1 in strawberry. (a) Phylogenetic relationship of protein sequences of STOP1 from strawberry and other plant species. The phylogenetic analysis was aligned using the neighbour‐joining method. The red circle represents FvSTOP1. (b) Amino acid sequence alignment of STOP1 from strawberry and other plant species. The sequences for alignment were the 255–417 aa region of the FvSTOP1. The amino acid sequence alignment was done using DNAMAN software. The red triangles represent the location of the zinc finger domains.
**Figure S2** The leaves and flowers phenotypes of overexpressing and knockout *FvSTOP1* transgenic strawberry plants. (a) The leaves and flowers phenotypes of wild‐type (‘Ruegen’, WT) and overexpressing *FvSTOP1* plants (FvSTOP1‐OE). (b) The leaves and flowers phenotype of WT and *FvSTOP1* knockout transgenic strawberry plants (*fvstop1*‐*cr*). Scale bar = 1 cm.
**Figure S3** Gene editing types of *FvSTOP1* knockout transgenic strawberry plants. sgRNA1 and sgRNA2 target sites were mutated in *fvstop1‐1*, *fvstop1‐2*, and *fvstop1‐3* strawberry plants. The dotted red line is the sgRNA position. The ellipsis represents the missing nucleotides. The red font indicates the inserted nucleotide. The yellow box is the intermediate sequence between sgRNA1 and sgRNA2. (a) The gene editing type of *fvstop1‐1* line (a 295 bp base inversion in the interval of sgRNA1 and sgRNA2). (b) Sequencing diagram of *fvstop1‐1*. (c) *fvstop1‐2* is a cross of *fvstop1‐1* and *fvstop1‐3*. (d) Sequencing diagram of *fvstop1‐2*. (e) *fvstop1‐3* line with a T nucleotide inserted at sgRNA1 to form the stop codon TGA and a nucleotide missing at sgRNA2. (f) Sequencing diagram of *fvstop1‐3*.
**Figure S4** The developmental phenotypes of WT and *fvstop1‐cr* 3# transgenic strawberry plants. The phenotypes of WT and *fvstop1‐cr* 3# transgenic strawberry plants at 30, 60, and 90 days after transplanting to the greenhouse. Scale bar = 1 cm.
**Figure S5** Flavonoid metabolomics analysis of WT and *fvstop1‐cr* 1#, *fvstop1‐cr* 2# knockout transgenic strawberry plants. (a) Volcano diagram of metabolic profiles in WT and *fvstop1‐cr* 1#. The red circles, green squares, and grey triangles represent the up‐regulated, down‐regulated, and insignificant metabolites, respectively. The horizontal axis represents the fold change of the metabolites content, and the vertical axis represents the significant level of the difference. (*P*‐value < 0.05 and |log_2_FC| > 0.58). (b) Differential altered metabolites in WT and *fvstop1‐cr* 1#. (c) Volcano diagram of metabolic profiles in WT and *fvstop1‐cr* 2#. The red circles, green squares, and grey triangles represent the up‐regulated, down‐regulated, and insignificant metabolites, respectively. The horizontal axis represents the fold change of the metabolites content, and the vertical axis represents the significant level of the difference. (*P*‐value < 0.05 and |log_2_FC| > 0.58). (d) Differential altered metabolites in WT and *fvstop1‐cr* 2#. (e) Differential altered metabolites of WT versus *fvstop1‐cr* 1# and WT versus *fvstop1‐cr* 2# on the Venn diagram. (f) Commonly shared down‐regulated metabolites of WT versus *fvstop1‐cr* 1# and WT vs *fvstop1‐cr* 2#. (*P*‐value < 0.05 and |log_2_FC| > 0.58). (g) Simplified scheme of flavonoid metabolic pathway in plants. The metabolites that were significantly regulated in *fvstop1‐cr* 1# and *fvstop1‐cr* 2# compared with WT are indicated in red font. Square visually represents the altered content levels of differential metabolites. (*P*‐value < 0.05 and |log_2_FC| > 0.58). 3GT, flavonoid‐3‐O‐glucosyltransferase; 4CL, 4‐coumarate CoA ligase; BAHD, acyl‐CoA‐dependent acyltransferases; C4H, cinnamate‐4‐hydroxylase; C‐Glc, C‐linked glycosylation; CHI, chalcone isomerase; CHS, chalcone synthase; COMT, catechol‐O‐methyltransferase; DFR, dihydroflavonol 4‐reductase; F2H, flavanone‐2‐hydroxylase; F3′5′H, flavonoid‐3′,5′‐hydroxylas; F3′H, flavonoid 3′‐hydroxylase; F3H, flavanone 3‐hydroxylase; FLS, flavonol synthase; FNS, flavone synthase; FOMT, flavonoid O‐methyltransferase; IFR, isoflavone reductase; IFS, isoflavone synthase; LAR, leucoanthocyanidin reductase; NS, anthocyanidin synthase; O‐Glc, O‐linked β‐N‐acetylglucosamine; OMT, O‐methyltransferase; PAL, phenylalanine ammonia lyase; PTS, pterocarpan synthase; UGT, UDP‐flavonoid glucosyl transferase.
**Figure S6** FvSTOP1 cannot bind to the promoters of *FvCHS*, *FvF3H*, *FvDFR*, *FvANS*, and *FvTT12* by yeast one‐hybrid (Y1H) assay. *FvCHS*, *FvF3H*, *FvDFR*, *FvANS*, and *FvTT12* promoter regions were segmented and ligated to the pAbAi vector. The pGADT7 vector was the control. The plasmids harbouring promoters‐pAbAi and FvSTOP1‐AD were transferred into Y1H yeast cells. Positive single colonies were selected and assigned to SD/−Leu and SD/−Leu/+AbA (25–500 μg/L) medium. (a–d) FvSTOP1 cannot bind to the promoters of *FvCHS*. (e) FvSTOP1 cannot bind to the promoters of *FvANS*. (f–h) FvSTOP1 cannot bind to the promoters of *FvF3H*. (i, j) FvSTOP1 cannot bind to the promoters of *FvTT12*. (k–n) FvSTOP1 cannot bind to the promoters of *FvDFR*. (o–p) FvSTOP1 cannot bind to the promoters of *FvTT19‐1* and *FvTT19‐2*. The segmentations are as follows: *proFvCHS* (*proFvCHS1‐1*: ‐1790‐‐1548 bp, *proFvCHS1‐2*: ‐857‐‐568 bp, *proFvCHS2*: ‐1570‐‐836 bp, *proFvCHS3*: ‐588‐‐1 bp), *proFvF3H* (*proFvF3H‐1*: ‐2001‐‐1032 bp, *proFvF3H‐2*: ‐1091‐‐608 bp, *proFvF3H‐3*: ‐646‐‐1 bp), *proFvDFR* (*proFvDFR‐1*: ‐2253‐‐1638 bp, *proFvDFR‐2*: ‐1698‐‐1042 bp, *proFvDFR‐3*: ‐1113‐‐463 bp, *proFvDFR‐4*: ‐552‐‐1 bp), *proFvANS* (‐2001‐‐1 bp), *proFvTT12* (*proFvTT12‐2*: ‐1056‐‐481 bp, *proFvTT12‐3*: ‐552‐‐1 bp), *proFvTT19* (*proFvTT19‐1*: ‐1455‐‐913 bp, *proFvTT19‐2*: ‐975‐‐479 bp, *proFvTT19‐3*: ‐534‐‐1 bp).
**Figure S7** The expression patterns of *FvTT19* in different organs of woodland strawberry. RT‐qPCR detected the expression patterns of *FvTT19* in different organs of woodland strawberry. Different letters indicated significant differences compared with the shoot apex. Data are the mean ± SD of three biological replicates with Tukey's hoc test (*P* < 0.05).
**Figure S8** Functional analysis of *FvTT19* in strawberry fruits by transient expression assay. (a) The *Agrobacterium* bacterial suspension harbouring *p35S*::*FvTT19* and *FvTT19‐*RNAi were constructed and injected into the green stage fruits (18 days after pollination) of the ‘Ruegen’, and at least 15 fruits (green stage) were selected for transient transformation. Scale bar = 1 cm. (b) Anthocyanin contents in transient transformation fruits. The circles represent the colour of the anthocyanin extract. FW is Fresh weight. Different letters represent significant differences compared with the control with Tukey's hoc test (*P* < 0.05). (c) Relative expression of anthocyanin‐related genes in *p35S*::*FvTT19* and *FvTT19‐*RNAi transient transformation fruits. Data are the mean ± SD of three biological replicates. Significant differences were analysed by Student's *t*‐test (*P < 0.05, **P < 0.01, ***P < 0.001).
**Figure S9** Relative expression of anthocyanin‐related genes in transient transformation fruits with different combinations. (a) Relative expression of anthocyanin‐related genes in co‐injection of FvMYB1‐RNAi and FvbHLH33‐RNAi plasmids into the green stage fruits (18 days after pollination) of the ‘Ruegen’. (b) Relative expression levels of anthocyanin‐related genes in co‐injection of FvMYB1‐RNAi and FvbHLH33‐RNAi plasmids into the green stage fruits of the ‘Ruegen’ and *fvstop1‐cr* 2# plants. Data are the mean ± SD of three biological replicates. Significant differences were analysed by Student's *t*‐test (*P < 0.05, **P < 0.01, ***P < 0.001).
**Figure S10** The coding sequence alignment between *FvTT19* and *RAP*.

## Data Availability

The data that support the findings of this study are available on request from the corresponding author. The data are not publicly available due to privacy or ethical restrictions.
