# Supplementary material for: A novel function for the transcription factor sensitive to proton rhizotoxicity1 in promoting anthocyanin accumulation in strawberry
Source: Plant Biotechnol J. 2025 Jun 10;23(9):3727–47. doi: 10.1111/pbi.70194 (PMC12392964; doi:10.1111/pbi.70194)
Supplement: Supplementary file 1 — File S1 The coding sequences of FvSTOP1 (XP_004290434.1), FvMYB1 (XM_004299494.2), FvbHLH33 (XM_004308329.2). [file PBI-23-3727-s001.docx]

**File S1.**

The coding sequences of *FvSTOP1* (XP_004290434.1), *FvMYB1* ([XM_004299494.2](https://www.ncbi.nlm.nih.gov/nucleotide/XM_004299494.2?report=genbank&log$=nucltop&blast_rank=1&RID=MY5EUMGD013)), *FvbHLH33* (*XM_004308329.2*)

**Coding sequences of** ***FvSTOP1***

ATGGATCATAAAGATAAGGTTTGTGCAGACACCTGGGCAAAGGATTTAAGAAATAAGACTTGCTCAGATCGGCCAAAGTTTGCCAATTTCAGTGCACACCAGCATCAAAACAAGTGGGAAGATCCCTCCATTTTAGATTATGGCATTAGGATTGAACCATCCTTCCAGAAACTCAGCCAGCCATCTGAGGATCAAACTTCACTTCCTCACAACTCCAACAATGAGAAAACAATTGCAGATGGGGAAGATGTTCAGATGAATGAGATATTTCACGCCAGTAAGATTCAAGATTGGGATCCGAGTACAATGTTGACCAATCTATCCTTCCTGGAACAAAAGATCCATCAGCTCCAAGATTTAGTGCATTTGATTGTTGGCCGGAGAGGACAAGTTCTAGGACGACCTGATGAGCTGGTGGCTCAACAACAGCAGCTCATTACTGCTGATCTTACTTCAATCATAGCTCAATTGATCTCTACGGCAGGTAGTCTTCTTCCATCCGTCAAGCATACCCTTTCCAGCGCCTCAGCATCTGCGGTACAGTTCGGGCAGCTTGGTGGTTCATTTGTTTCTTCTGGAGCAGGAACTGAAGCTTCTGTTAAGTTGCAAATGAATTGCGGAAGCAAGTTACCTGAGCAGCCCAACCAAACTGACCCGATCAGTAACTATGGGACTGAGCTAAACTACCACATTGAAGAACATGAATCAAAAGATGAAGAGGATGCTGATGAAGGCGAGAACCTTCCACCTGGTACTTACGAAATCTTACAGCTAGAGAAAGAAGAAATCCTTGCACCACATACTCATTTCTGTGCTATATGCGGAAAGGGATTCAAGAGGGATGCAAACTTAAGGATGCACATGAGAGGTCATGGAGACGAGTACAAAACCCCAGCTGCACTTGCAAAGCCCAACAAAGATTCTAGTTCTGAACCAACACTCATCAAAAGGTATTCATGCCCTTATGCTGGTTGCAAGCGGAACAAGGATCACAAGAAGTTTCAGCCTTTGAAGACCATTTTATGTGTCAAAAATCACTACAAGAGAACCCATTGTGACAAAAGTTACACTTGCAGCAGATGCAACACCAAGAAGTTCTCCGTTATTGCAGATCTCAAGACTCATGAGAAGCACTGTGGTAAGGACAAGTGGCTTTGTTCTTGTGGCACAACCTTCTCCAGGAAAGACAAGCTTTTTGGCCATATTGCCCTGTTCCAAGGTCACACTCCTGCCATTCCCCTCGATGAAACAAAAACTGCTGTTGGGGCATCTGAACATGGGGAAGGTAGTGAAGCACCAAACAGAGTTGGTAGCATCAATTTCAATTTTGGTTCCACTCTTCCAAGTGCCGGTGGTTTGGTTCAGAATATTATGGATGTGAAAGAAAGTGTCGACGATCCTACTTGTTATTTCTCGCCGTTGAATTTTGACACATGTAATTTTGATGGATTTCATGAGTTCCCTCGACCCCCATTTGAAGATTCAGAGAGTTCATTCTCTTTTCTCATGCCAGGGTCCTGTAATTACACTCACAAAACTGGAGGCGAGTCAAATTCCAAACAGGTCGAGTGA

**Coding sequences of** ***FvMYB1***

ATGAGGAAGCCCTGCTGCGAGAAGACGGAGACGACTAAAGGGGCGTGGTCGATCCAAGAAGATCAGAAACTCATTGACTACATCCAAAAACACGGCGAAGGTTGCTGGAATTCGCTTCCTAAGGCTGCAGGGTTGCGTCGTTGTGGTAAGAGTTGTCGACTGAGATGGATAAACTATCTACGACCTGATCTTAAACGAGGGAGCTTTAGTGAAGATGAAGAGGATCTCATCATCAGGCTTCATAAACTCCTTGGGAATAGGTGGTCGCTAATAGCTGGAAGACTGCCTGGAAGGACAGATAACGAAGTGAAGAACTACTGGAACTCTCATTTAAAGAAGAAGATACTGAACACAGGCACTACTCTTCGTCCAAATAAGCCCCGCCCTGAGATTAAGCATGCACCTTATAACAAACTTGTCAAGTACTTCAATGAGATGGACGATGAGGTCGTTGATGAGGTCTCATCAGCCGATTCTGCTGCTGGCTGTTTGGTGCCTGAGTTGAATCTCGACCTCACTTTAAGCATCAAGACTAGTACTGGAATGGCTGATCCTCAAGTTGCTTAA

**Coding sequences of** ***FvbHLH33***

ATGGCCAATGGGACTCAAATCCATGAGAGGGTGCCTGAGAATCTGAGGAAACAGTTTGCTGTTGCTGTGAGGAGTATTAAGTGGAGCTATGCAATTTTCTGGTCATTGTCAACAACTCAACAAGGGGTGCTGGAATGGGGTGAAGGGTATTACAATGGAGACATCAAAACCAGGAAGACGGTCGAAGGAATAGAACCTAAAGCTGATAAAATAGGTTTGCAGAGGAATGAACAACTTAGAGAGTTGTACAAGGCTCTCTTAGAAGGTGAGTCGACAGATCAACAAGCCAAAGTGCCTTCTGCTGCATTGTCTCCGGAGGATCTCACGGATGCGGAGTGGTATTACTTGCTTTGCATGTCATTCGTGTTCAATATTGGCGAAGGTCTGCCAGGAAGAGCATTAGCAAACGAGAAAAGCATTTGGTTATGCAATGCTCAATATGCAGATAGCAAAGTATTTTCTCGTTCTTTGCTAGCAAAGAGTGCTTCTATTCAGACTGTGGTCTGCTTTCCCTATCTTGGAGGTGTTGTTGAGCTAGGTGTCACGGAGCTGGTAGCGGAGGACCCTAGTCTCCTTCAACACATCAAGGCTTCCTTACTAGATTTCTCAAAGCCAGATTGCTCCGAGAAATCTTCCTCTGCTCCTCACAAAGCAGATGATGATTCAGACCAATTTCTTGCCAACATTGTAGGCCATGAAATAGTTGATACATTGGGTTTGGAGAACCTGTATTCCCCTTCACAAGAACTCAAATTTGATCAGAGTGGAATTAATGGATTACGTGGACATGATGAAGAGTTCAGCATGGGATCTGCTGATGAATGTTCCAAGGGATGTGAGCACAATCATCAGACAGATGACTCCTTTATGCTTGAAGCTGTCAATGGTGGGGCTTCTCAAGTTCAGAGTTGGCATTTCATGGATGATGATTTCAGCAATGGATTTCAAGATTCCATGAATTCTAGTGATTGTATATCAGAAGCTTTCGTGGGAAAGGCTCATTCTTCTACTATACCTGAGAATAAGAATCATAGTCATTTAAAAGAACTTCAGAACTTCAATGATACAAAGTTCAGCTCCTTGGGTCTTGGACCAGCTGATGATCATTTACACTACAAACAAACTGTTTCTGCTATTCTGGGGAGCTCGATGCGGATAATTGGAAACCCATGCTTTTGCAGTGGAGATAGAAAATCTAGTTTCGTGCAATGGACAAAAGGTGCTGTTCATTATTGTAGGCCAAAAGCTCAACAGAAACTGTTAAAGAAGATTTTGTTTACAGTTCCACTGATGATGAGTGGTGGCTCTCCTAGTCCACAAAAAGAATCTACTACAAACTCCAAATCAGAAAGTGATGATGTGCACGAGAAATTGATAGAGAATGAGAAGTTGATGGTTCTGAGGTCAATGGTTCCTTCTATGACTGAGATTGACAAGGCATCGATCCTCGATGATACAATTAAGTACTTGAAAGAGCTTGAGGCAAGAGCAGAAGAGATGGAATCTTGCATGGACACTGTGGAAGCAATAGCTAGAAGGAAGTTCCTAGATAGGGTAAAGAAGGCATCAGATAACAAAACAAAGACTGGCAATGCTAAAAAGCCTTCGATAAACAAGCGGAAGGCCTGTGACATTGATGAAACTGACCCGGAACTTAATAGGCTTGTCTCCAAAGAAAGCTTACCACTCGATGTGAATGTCAGTGTAAAAGAGCAGGAGGTTCTGATAGAGATGAAATGCCCTTATAGGGAATACATCTTGCTTGATATAATGGATGCTGTTAACAGTCTGTACTTGGATGCTCACTCAGTGCAATCATCCACCCTCAATGATGTTCTCACATTGAGCCTTAAATCAAAGTTTCGAGGATCAGCAATCGCACCGGTGGGGATGATAAAACAAGCGCTTTGGAAAATTGCTGGTAAGTGTTAA
