# Supplementary material for: A novel function for the transcription factor sensitive to proton rhizotoxicity1 in promoting anthocyanin accumulation in strawberry
Source: Plant Biotechnol J. 2025 Jun 10;23(9):3727–47. doi: 10.1111/pbi.70194 (PMC12392964; doi:10.1111/pbi.70194)
Supplement: Supplementary file 2 — Table S1 Primer sets were used in this study. [file PBI-23-3727-s003.docx]

**Supplemental information**

**Supplemental Table**

**Supplemental Table S1.** Primer sets were used in this study.

| **Primers for RT-qPCR assay** | | | | | | | | | |
| --- | --- | --- | --- | --- | --- | --- | --- | --- | --- |
| Fv26S-F | | TAACCGCATCAGGTCTCCAA | | | | | | | |
| Fv26S-R | | CTCGAGCAGTTCTCCGACAG | | | | | | | |
| FvSTOP1-qPCR-F | | CGGAAGCAAGTTACCTGAGC | | | | | | | |
| FvSTOP1-qPCR-R | | TTTGCATCCCTCTTGAATCC | | | | | | | |
| FvMYB1-qPCR-F | | TTGCGTCGTTGTGGTAAGAG | | | | | | | |
| FvMYB1-qPCR-R | | TCTGTCCTTCCAGGCAGTCT | | | | | | | |
| FvbHLH33-qPCR-F | | GTTCCAAGGGATGTGAGCAC | | | | | | | |
| FvbHLH33-qPCR-R | | AGAATGAGCCTTTCCCACGA | | | | | | | |
| FvCHS-qPCR-F | | GACATACCTGGTGCCGACTT | | | | | | | |
| FvCHS-qPCR-R | | AGTGCCTGTCCGACCAATAC | | | | | | | |
| FvF3H-qPCR-F | | AGGGTGGCTTCATCGTTTC | | | | | | | |
| FvF3H-qPCR-R | | GGCTGGGGGCATTTCGGGT | | | | | | | |
| FvDFR-qPCR-F | | CTGGAGCGATGTCGAATTTT | | | | | | | |
| FvDFR-qPCR-R | | AGAGGCGAAAGTCCGGTAAT | | | | | | | |
| FvANS-qPCR-F | | CCTCAAACACCTTCCGACT | | | | | | | |
| FvANS-qPCR-R | | CCTCCCTTCTTCTAATCCC | | | | | | | |
| FvTT12-qPCR-F | | GCAGAGGTTCCTTCAAGCAC | | | | | | | |
| FvTT12-qPCR-R | | AAGCAACCACCAGGACAAAC | | | | | | | |
| FvTT19-qPCR-F | | ACTTGTGATCCTTCCCAGCA | | | | | | | |
| FvTT19-qPCR-R | | TCAACCAGAAACCGAATCGC | | | | | | | |
| **Primers for subcellular localization assay** | | | | | | | | | |
| FvSTOP1-GFP-SalⅠ-F | | | ACTGTTGATACATATGCCCGTCGACATGGATCATAAAGATAAGGT | | | | | | |
| FvSTOP1- GFP-BamHⅠ-R | | | CCTCGCCCTTGCTCACCATGAATCCCTCGACCTGTTTGGAATT | | | | | | |
| **Primers for transient and stable transformation assay** | | | | | | | | | |
| FvSTOP1- pRI101-NdeⅠ-3Flag-F | | | | | | | GCACATATGGACTACAAAGACCATGACGGTGATTATAAAGATCATGACATCGACTACAAGGATGACGATGACAAGATGGATCATAAAGATAAGGTTT | | |
| FvSTOP1- pRI101-EcoRⅠ-R | | | | | | | GCAGAATTCTCACTCGACCTGTTTGGAA | | |
| FvSTOP1-RNAi-XbaⅠ-forward-F | | | | | | | AGAGAACACGGGGGACTCTAGAATGTTGACCAATCTATCCTT | | |
| FvSTOP1-RNAi- forward-SalⅠ-R | | | | | | | TTGGGGTTCCCCCGGGGTCGACCTTAACAGAAGCTTCAGTTC | | |
| FvSTOP1-RNAi-reverse-BamHⅠ-F | | | | | | | CGAAATCGATGGTACCGGATCCCTTAACAGAAGCTTCAGTTC | | |
| FvSTOP1-RNAi-reverse-SacⅠ-R | | | | | | | ACGATCGGGGAAATTCGAGCTCATGTTGACCAATCTATCCTT | | |
| FvSTOP1- AtU3dT1 | | | | | | | CAGATGGCTGGCTGAGTTTCTGACCAATGGTGCTTTG | | |
| FvSTOP1- gRT1 | | | | | | | GAAACTCAGCCAGCCATCTGGTTTTAGAGCTAGAAAT | | |
| FvSTOP1- AtU3dT2 | | | | | | | GCCGTAGAGATCAATTGAGCTGACCAATGGTGCTTTG | | |
| FvSTOP1- gRT2 | | | | | | | GCTCAATTGATCTCTACGGCGTTTTAGAGCTAGAAAT | | |
| sgRNA-F | | | | | | | ATGGATCATAAAGATAAGGT | | |
| FvTT19- pRI101-NdeI -F | | | | | CAAGTTCTTCACTGTTGATACATATGATGGTACTGAAAGTTTATGG | | | | |
| FvTT19- pRI101-BamHI-R | | | | | GAGTTGTTGATTCAGAATTCGGATCCCTAGTATTGAGCAAGCTTCA | | | | |
| FvTT19- RNAi-NdeⅠ- forward-F | | | AGAGAACACGGGGGACCATATGATGGTACTGAAAGTTTATGGTCCAG | | | | | | |
| FvTT19-RNAi-SalⅠ-forward-R | | | TTGGGGTTCCCCCGGGGTCGACGAGCCATTGATCCACCAGTGCCTTC | | | | | | |
| FvTT19-RNAi-BamHI-reverse-F | | | CGAAATCGATGGTACCGGATCCGAGCCATTGATCCACCAGTGCCTTC | | | | | | |
| FvTT19-RNAi-SacⅠ-reverse-R | | | ACGATCGGGGAAATTCGAGCTCATGGTACTGAAAGTTTATGGTCCAG | | | | | | |
| FvMYB1-RNAi -XbaI- forward-F | | | GCATCTAGAATAAACTCCTTGGGAATAGGT | | | | | | |
| FvMYB1-RNAi -SmaI- forward-R | | | GCACCCGGGTTAAGCAACTTGAGGATCAGC | | | | | | |
| FvMYB1-RNAi-KpnI- reverse-F | | | GCAGGTACCTTAAGCAACTTGAGGATCAGC | | | | | | |
| FvMYB1-RNAi-SacI- reverse-R | | | GCAGAGCTCATAAACTCCTTGGGAATAGGT | | | | | | |
| FvbHLH33-RNAi-XbaⅠ forward-F | | | AGAGAACACGGGGGACTCTAGATCTGAGGAAACAGTTTGCTGTTGCT | | | | | | |
| FvbHLH33-RNAi-forward SmaI-R | | | TACCAATTGGGGTTCCCCCGGGTACCACTCCGCATCCGTGAGATCCT | | | | | | |
| FvbHLH33-RNAi-KpnⅠ-reverse-F | | | CGAAATCGATGGTACCGGATCCTACCACTCCGCATCCGTGAGATCCT | | | | | | |
| FvbHLH33-RNAi-SacI- revers-R | | | ACGATCGGGGAAATTCGAGCTCTCTGAGGAAACAGTTTGCTGTTGCT | | | | | | |
| **Primers for transcriptional activation assay** | | | | | | | | | |
| FvSTOP1-BD-EcoRⅠ-F | | | | | | | | | GCAGAATTCATGGATCATAAAGATAAGGTT |
| FvSTOP1- BD-PstⅠ-F | | | | | | | | | GCACTGCAGTCACTCGACCTGTTTGGAA |
| FvSTOP1-N1-BD-BamHⅠ-F | | | | | | | | | GGCCGAATTCCCGGGGATCCATGGATCATAAAGATAAGGTT |
| FvSTOP1-N1-BD-SalⅠ-R | | | | | | | | | TGCGGCCGCTGCAGGTCGACTGTACTCGGATCCCAATCT |
| FvSTOP1-N2-BD-BamHⅠ-F | | | | | | | | GGCCGAATTCCCGGGGATCCATGTTGACCAATCTATCCTTC | |
| FvSTOP1-N2-BD-SalⅠ-R | | | | | | | | TGCGGCCGCTGCAGGTCGACAGTACCAGGTGGAAGGTTC | |
| FvSTOP1-C2H2-BD-BamHⅠ-F | | | | | | | | GGCCGAATTCCCGGGGATCCTACGAAATCTTACAGCTAGAG | |
| FvSTOP1-C2H2-BD- SalⅠ-R | | | | | | | | TGCGGCCGCTGCAGGTCGACGGGAATGGCAGGAGTGTG | |
| FvSTOP1-C-BD-BamHⅠ-F | | | | | | | | GGCCGAATTCCCGGGGATCCCTCGATGAAACAAAAACTGCT | |
| FvSTOP1-C-BD-SalⅠ-R | | | | | | | | TGCGGCCGCTGCAGGTCGACTCACTCGACCTGTTTGGAAT | |
| FvbHLH33-BD-BamHⅠ-F | | | | | | | | GGCCGAATTCCCGGGGATCCATGGCCAATGGGACTCAAAT | |
| FvbHLH33-BD-SalⅠ-R | | | | | | | TGCGGCCGCTGCAGGTCGACTTAACACTTACCAGCAATTT | | |
| FvbHLH33-bHLH-MYC-N-BD-BamHⅠ-F | | | | | | | GGCCGAATTCCCGGGGATCCATGGCCAATGGGACTCAAAT | | |
| FvbHLH33-bHLH-MYC-N-BD-SalⅠ-R | | | | | | | TGCGGCCGCTGCAGGTCGACAGCCTTGATGTGTTGAAGGA | | |
| FvbHLH33-Center-BD-BamHⅠ-F | | | | | | | GGCCGAATTCCCGGGGATCCTCCTTACTAGATTTCTCAAA | | |
| FvbHLH33-Center-BD-SalⅠ-R | | | | | | | TGCGGCCGCTGCAGGTCGACGTGCACATCATCACTTTCTG | | |
| FvbHLH33-bHLH-SF-BD-BamHⅠ-F | | | | | | | GGCCGAATTCCCGGGGATCCGAGAAATTGATAGAGAATGA | | |
| FvbHLH33-bHLH-SF-BD-SalⅠ-R | | | | | | | TGCGGCCGCTGCAGGTCGACTTAACACTTACCAGCAATTT | | |
| **Primers for yeast one-hybrid, LUC, GUS assay** | | | | | | | | | |
| FvSTOP1-AD-EcoRⅠ-F | | | | | GCAGAATTCATGGATCATAAAGATAAGGTT | | | | |
| FvSTOP1-AD-PstⅠ-R | | | | | GCACTGCAGTCACTCGACCTGTTTGGAA | | | | |
| FvSTOP1-N1-AD-EcoRⅠ-F | | | | | CATATGGCCATGGAGGCCAGTGAATTCATGGATCATAAAGATAAGGTT | | | | |
| FvSTOP1-N1-AD-BamHⅠ-R | | | | | ATCTGCAGCTCGAGCTCGATGGATCCTGTACTCGGATCCCAATCT | | | | |
| FvSTOP1-N2-AD-EcoRⅠ-F | | | | | CATATGGCCATGGAGGCCAGTGAATTCATGTTGACCAATCTATCCTTC | | | | |
| FvSTOP1-N2-AD-BamHⅠ-R | | | | | ATCTGCAGCTCGAGCTCGATGGATCCAGTACCAGGTGGAAGGTTC | | | | |
| FvSTOP1-C2H2-AD-EcoRⅠ-F | | | | | CATATGGCCATGGAGGCCAGTGAATTCTACGAAATCTTACAGCTAGAG | | | | |
| FvSTOP1-C2H2-AD-BamHⅠ-R | | | | | ATCTGCAGCTCGAGCTCGATGGATCCGGGAATGGCAGGAGTGTG | | | | |
| FvSTOP1-C-AD-EcoRⅠ-F | | | | | CATATGGCCATGGAGGCCAGTGAATTCCTCGATGAAACAAAAACTGCT | | | | |
| FvSTOP1-C-AD-BamHⅠ-R | | | | | ATCTGCAGCTCGAGCTCGATGGATCCTCACTCGACCTGTTTGGAAT | | | | |
| FvbHLH33-bHLH-MYC-N-AD-EcoRⅠ-F | | | | | | CATATGGCCATGGAGGCCAGTGAATTCATGGCCAATGGGACTCAAAT | | | |
| FvbHLH33-bHLH-MYC-N-AD-BamHⅠ-R | | | | | | ATCTGCAGCTCGAGCTCGATGGATCCAGCCTTGATGTGTTGAAGGA | | | |
| FvbHLH33-Center-AD-EcoRⅠ-F | | | | | | CATATGGCCATGGAGGCCAGTGAATTCTCCTTACTAGATTTCTCAAA | | | |
| FvbHLH33-Center-AD-BamHⅠ-R | | | | | | ATCTGCAGCTCGAGCTCGATGGATCCGTGCACATCATCACTTTCTG | | | |
| FvbHLH33-bHLH-SF-AD-EcoRⅠ-F | | | | | | CATATGGCCATGGAGGCCAGTGAATTCGAGAAATTGATAGAGAATGA | | | |
| FvbHLH33-bHLH-SF-AD-BamHⅠ-R | | | | | | ATCTGCAGCTCGAGCTCGATGGATCCTTAACACTTACCAGCAATTT | | | |
| FvMYB1-bHLH-BD-BamHⅠ-F | | | | | | GGCCGAATTCCCGGGGATCCATGAGGAAGCCCTGCTGCGA | | | |
| FvMYB1-bHLH-BD- SalⅠ-R | | | | | | TGCGGCCGCTGCAGGTCGACCTTCTTCTTTAAATGAGAGT | | | |
| FvMYB1-EAR-BD-BamHⅠ-F | | | | | | GGCCGAATTCCCGGGGATCCATACTGAACACAGGCACTACT | | | |
| FvMYB1-EAR-BD- SalⅠ-R | | | | | | TGCGGCCGCTGCAGGTCGACTTAAGCAACTTGAGGATCAG | | | |
| *ProFvCHS1-1*- pAbAi-SacⅠ-F | | | | | GCAGAGCTCGATCACGTGCCTGCCCAATT | | | | |
| *proFvCHS1-1*- pAbAi-SalⅠ-R | | | | | GCAGTCGACTGCAAATACATGTGTAATGTCG | | | | |
| *ProFvCHS1-2*- pAbAi-SacⅠ-F | | | | | GCAGAGCTCCTCCAAGCAGATATTTATAGCA | | | | |
| *proFvCHS1-2*- pAbAi-SalⅠ-R | | | | | GCAGTCGACGTTTGTTGGGACACTTGCTG | | | | |
| *ProFvCHS2*-pAbAi-HindⅢ-F | | | | | AAATGATGAATTGAAAAGCTTCGACATTACACATGTATTTGCA | | | | |
| *proFvCHS2*-pAbAi-SacⅠ-R | | | | | AGATCCCCGGGTACCGAGCTCTGCTATAAATATCTGCTTGGAG | | | | |
| *proFvCHS3*-pAbAi-HindⅢ-F | | | | | AAATGATGAATTGAAAAGCTTCAGCAAGTGTCCCAACAAAC | | | | |
| *proFvCHS3*-pAbAi-SacⅠ-R | | | | | AGATCCCCGGGTACCGAGCTCCTGTGGTGGATTTTGAAATT | | | | |
| *proFvF3H-1*-pAbAi-HindⅢ-F | | | | | AAATGATGAATTGAAAAGCTTTTCTGATATATGCTCTCTA | | | | |
| *proFvF3H*-pAbAi-SacⅠ-F | | | | | GCAGAGCTCAGAAGCAGGAGATTGGCATG | | | | |
| *proFvF3H*-pAbAi-SmaⅠ-R | | | | | GCACCCGGGCTCTCTACACCTAAACCTTGT | | | | |
| *proFvF3H-1*-pAbAi-SacⅠ-R | | | | | AGATCCCCGGGTACCGAGCTCTTGTTAGGTGAATATCACAT | | | | |
| *proFvF3H-*3-pAbAi-HindⅢ-F | | | | | AAATGATGAATTGAAAAGCTTGGATTTAAGTTGCTTAATAC | | | | |
| *proFvF3H-*3-pAbAi-SacⅠ-R | | | | | AGATCCCCGGGTACCGAGCTCATGGAGTGTGTTAATAACA | | | | |
| *ProFvDFR-1*-pAbAi-HindⅢ-F | | | | | AAATGATGAATTGAAAAGCTTAGAACAAGCCGCCAACAGC | | | | |
| *ProFvDFR-1*-pAbAi-SacⅠ-R | | | | | AGATCCCCGGGTACCGAGCTCAGACATGATAGACATGAAG | | | | |
| *ProFvDFR-2*-pAbAi-HindⅢ-F | | | | | AAATGATGAATTGAAAAGCTTAAGCCTGTCAGTGTCACAA | | | | |
| *ProFvDFR-2*-pAbAi-SacⅠ-R | | | | | AGATCCCCGGGTACCGAGCTCGAAGCCAAGGACACCAGTC | | | | |
| *ProFvDFR-3*-pAbAi-HindⅢ-F | | | | | AAATGATGAATTGAAAAGCTTTGTAGTAGCCTTTCACAAC | | | | |
| *ProFvDFR-3*-pAbAi-SacⅠ-R | | | | | AGATCCCCGGGTACCGAGCTC TTAGACACACAAGTGCAAC | | | | |
| *ProFvDFR-4*-pAbAi-HindⅢ-F | | | | | AAATGATGAATTGAAAAGCTTATATTGAAGAAGCAGAAGA | | | | |
| *ProFvDFR-4*-pAbAi-SacⅠ-R | | | | | AGATCCCCGGGTACCGAGCTCTTTTACCCATCAAGTAATT | | | | |
| *ProFvANS*-pAbAi-HindⅢ-F | | | | | AAATGATGAATTGAAAAGCTTATGCATTTTAGAGCATGTA | | | | |
| *ProFvANS*-pAbAi-SacⅠ-R | | | | | AGATCCCCGGGTACCGAGCTCTGTCGCTTCTAGCTATAGC | | | | |
| *ProFvTT12-2*-pAbAi-HindⅢ-F | | | | | AAATGATGAATTGAAAAGCTTGTTATTATAATTAGGGTTA | | | | |
| *ProFvTT12-2*-pAbAi-SacⅠ-R | | | | | AGATCCCCGGGTACCGAGCTCACACATGCACTAATGCAGT | | | | |
| *ProFvTT12-3*-pAbAi-HindⅢ-F | | | | | AAATGATGAATTGAAAAGCTTTAGCCACACTCGATCAAAA | | | | |
| *ProFvTT12-3*-pAbAi-SacⅠ-R | | | | | AGATCCCCGGGTACCGAGCTCGGTTTCCAGTTGGTAAGGC | | | | |
| *ProFvTT19-1*-pAbAi-HindⅢ-F | | | | | AAATGATGAATTGAAAAGCTTCACTTCGGTCGAAACTTTT | | | | |
| *ProFvTT19-1*-pAbAi-SacⅠ-R | | | | | AGATCCCCGGGTACCGAGCTCTTTGAGGAAGGAGATTCAG | | | | |
| *ProFvTT19-2*-pAbAi-HindⅢ-F | | | | | AAATGATGAATTGAAAAGCTTCTCAATGACTGAGTCCAGA | | | | |
| *ProFvTT19-2*-pAbAi-SacⅠ-R | | | | | AGATCCCCGGGTACCGAGCTCCCAGAATGGAGAAGCAGT | | | | |
| *ProFvTT19-3*-pAbAi-HindⅢ-F | | | | | AAATGATGAATTGAAAAGCTTAAACATCACCGCCCCACCA | | | | |
| *ProFvTT19-3*-pAbAi-SacⅠ-R | | | | | AGATCCCCGGGTACCGAGCTCTTCGTACGTATATATTGCC | | | | |
| FvMYB1- pRI101-NdeI -F | | | | | CAAGTTCTTCACTGTTGATACATATGATGAGGAAGCCCTGCTGCGA | | | | |
| FvMYB1- pRI101-BamHI-R | | | | | GAGTTGTTGATTCAGAATTCGGATCCTTAAGCAACTTGAGGATCAG | | | | |
| FvbHLH33- pRI101-NdeI -F | | | | | CAAGTTCTTCACTGTTGATACATATATGGCCAATGGGACTCAAAT | | | | |
| FvbHLH33- pRI101-BamHI-R | | | | | GAGTTGTTGATTCAGAATTCGGATCTAACACTTACCAGCAATTT | | | | |
| *ProFvCHS*-LUC-HindⅢ-F | | | | | CGACGGTATCGATAAGCTTGATCACGTGCCTGCCCAATT | | | | |
| *ProFvCHS*-LUC-BamHl-R | | | | | CTCTAGAACTAGTGGATCCCTGTGGTGGATTTTGAAATT | | | | |
| *ProFvF3H*-LUC-HindⅢ-F | | | | | CGACGGTATCGATAAGCTTGGATTTAAGTTGCTTAATAC | | | | |
| *ProFvF3H*-LUC-BamHl-R | | | | | CTCTAGAACTAGTGGATCCATGGAGTGTGTTAATAACA | | | | |
| *ProFvTT19-3*-LUC-HindⅢ-F | | | | | CGACGGTATCGATAAGCTTCACTTCGGTCGAAACTTTT | | | | |
| *ProFvTT19-3*-LUC-BamHl-R | | | | | CTCTAGAACTAGTGGATCCTTCGTACGTATATATTGCC | | | | |
| *ProCHS*-201GUS-PstI-F | | | | | CCAGTGCCAAGCTTGCATGCCTGCAGGATCACGTGCCTGCCCAAT | | | | |
| *ProCHS*-201GUS-XbaI-R | | | | | TTTTGTGTGATTGTGATGTATCTAGACTGTGGTGGATTTTGAAATT | | | | |
| *ProF3H*-201GUS-PstI-F | | | | | CCAGTGCCAAGCTTGCATGCCTGCAGGGATTTAAGTTGCTTAATAC | | | | |
| ProF3H-201GUS-XbaI-R | | | | | TTTTGTGTGATTGTGATGTATCTAGAATGGAGTGTGTTAATAACA | | | | |
| *ProTT19-3*-201GUS-PstI-F | | | | | CCAGTGCCAAGCTTGCATGCCTGCAGAAACATCACCGCCCCACCA | | | | |
| *ProTT19-3*-201GUS-XbaI-R | | | | | TTTTGTGTGATTGTGATGTATCTAGATTCGTACGTATATATTGCC | | | | |
| **Primers for yeast two-hybrid, LCI, BiFC, pull-down assay** | | | | | | | | | |
| FvMYB1-AD-EcoRⅠ-F | | | CATATGGCCATGGAGGCCAGTGAATTCATGAGGAAGCCCTGCTGCGA | | | | | | |
| FvMYB1-AD-BamHⅠ-R | | | | ATCTGCAGCTCGAGCTCGATGGATCCTTAAGCAACTTGAGGATCAG | | | | | |
| FvSTOP1-Nluc-KpnⅠ-F | | | | AGAACACGGGGGACGAGCTCGGTACCATGGATCATAAAGATAAGGTTTGTG | | | | | |
| FvSTOP1-Nluc-SalⅠ-R | | | | CGGGACGCGTACGAGATCTGGTCGACCTCGACCTGTTTGGAATTTGACTCG | | | | | |
| FvMYB1- CLuc-KpnⅠ-F | | | | TCGTACGCGTCCCGGGGCGGTACCATGAGGAAGCCCTGCTGCGA | | | | | |
| FvMYB1-Cluc-BamHI-R | | | | GCGCGCCGGGCCCTCTAGAGGATCCTTAAGCAACTTGAGGATCAG | | | | | |
| FvbHLH33-CLuc-KpnⅠ-F | | | | TCGTACGCGTCCCGGGGCGGTACCATGGCCAATGGGACTCAAAT | | | | | |
| FvbHLH33-CLuc-BamHI-R | | | | GCGCGCCGGGCCCTCTAGAGGATCCTTAACACTTACCAGCAATTT | | | | | |
| FvSTOP1-pSPYCE-BamHI-F | | | | GGCCGCCACTAGTGGATCCATGGATCATAAAGATAAGGT | | | | | |
| FvSTOP1-pSPYCE-KpnI-R | | | | CATCCCGGGAGCGGTACCCTCGACCTGTTTGGAATTTGAC | | | | | |
| FvbHLH33-pSPYNE-BamHI-F | | | | GGCCGCCACTAGTGGATCCATGGCCAATGGGACTCAAAT | | | | | |
| FvbHLH33-pSPYNE-KpnI-R | | | | CATCCCGGGAGCGGTACCACACTTACCAGCAATTTTCC | | | | | |
| FvSTOP1-pSPYNE-BamHI-F | | | | GGCCGCCACTAGTGGATCCATGGATCATAAAGATAAGGT | | | | | |
| FvSTOP1- pSPYCE -KpnI-R | | | | CATCCCGGGAGCGGTACCCTCGACCTGTTTGGAATTTGAC | | | | | |
| FvSTOP1-His-KpnⅠ-F | | | ATCGAAGGTAGGCATATGGAGCTCGGTACCATGGATCATAAAGATAAGGT | | | | | | |
| FvSTOP1-His-EcoRⅠ-R | | | AGACTGCAGGTCGACAAGCTTGAATTCTCACTCGACCTGTTTGGAAT | | | | | | |
| FvbHLH33-His-BamHI-F | | | CATATGGAGCTCGGTACCCTCGAGGGATCCATGGCCAATGGGACTCAAAT | | | | | | |
| FvbHLH33-His -HindⅢ -R | | | TTACCTATCTAGACTGCAGGTCGACAAGCTTTTAACACTTACCAGCAATTT | | | | | | |
| FvMYB1-His-BamHI-F | | | CATATGGAGCTCGGTACCCTCGAGGGATCCATGAGGAAGCCCTGCTGCGA | | | | | | |
| FvMYB1-His-HindⅢ-R | | | TTACCTATCTAGACTGCAGGTCGACAAGCTTTTAAGCAACTTGAGGATCAG | | | | | | |
| MYB1-GST-BamHI-F | | | CCTCCAAAATCGGATCTGGTTCCGCGTGGATCCATGAGGAAGCCCTGCTGCGA | | | | | | |
| MYB1-GST-EcoRI-R | | | CACGATGCGGCCGCTCGAGTCGACCCGGGAATTCTTAAGCAACTTGAGGATCAG | | | | | | |
| FvSTOP1-GST-BamHI -F | | | CCTCCAAAATCGGATCTGGTTCCGCGTGGATCCATGGATCATAAAGATAAGGT | | | | | | |
| FvSTOP1-GST-EcoRI-R | | | CACGATGCGGCCGCTCGAGTCGACCCGGGAATTCTCACTCGACCTGTTTGGAAT | | | | | | |
| FvbHLH33-GST-BamHI-F | | | CCTCCAAAATCGGATCTGGTTCCGCGTGGATCCATGGCCAATGGGACTCAAAT | | | | | | |
| FvbHLH33-GST-EcoRI-R | | | CACGATGCGGCCGCTCGAGTCGACCCGGGAATTCTTAACACTTACCAGCAATTTTCC | | | | | | |
| **Primers for yeast three-hybrid assay** | | | | | | | | | |
| FvMYB1-MCS1-pBri-EcoRI-F  FvMYB1-MCS1-pBri-BamHI-R | GACAGTTGACTGTATCGCCGGAATTCATGAGGAAGCCCTGCTGCGA  TAGCTTGGCTGCAGGTCGACGGATCCTTAAGCAACTTGAGGATCAGGGATCCGTCGACCTGCAGCCAAGCTA | | | | | | | | |
| FvSTOP1-MCS2-pBri-NotI-F  FvSTOP1-MCS2-pBri-BglⅡ-R | TAAGAAGAAGAGAAAGGTGGCGGCCGCATGGATCATAAAGATAAGGT  GACATGGGAGATCAGCCCGAAGATCTTCACTCGACCTGTTTGGAAT  AGATCTTCGGGCTGATCTCCCATGTC | | | | | | | | |
| FvbHLH33-AD-ECORⅠ-F | CATATGGCCATGGAGGCCAGTGAATTCATGGCCAATGGGACTCAAAT | | | | | | | | |
| FvbHLH33-BamHⅠ-AD-R | ATCTGCAGCTCGAGCTCGATGGATCCTTAACACTTACCAGCAATTT | | | | | | | | |
