# Supplementary material for: A novel function for the transcription factor sensitive to proton rhizotoxicity1 in promoting anthocyanin accumulation in strawberry
Source: Plant Biotechnol J. 2025 Jun 10;23(9):3727–47. doi: 10.1111/pbi.70194 (PMC12392964; doi:10.1111/pbi.70194)
Supplement: Supplementary file 3 — Figure S1 Phylogenetic and structural analyses of FvSTOP1 in strawberry. (a) Phylogenetic relationship of protein sequences of STOP1 from strawberry and other plant species. The phylogenetic analysis was aligned using the neighbour‐joining method. The red circle represents FvSTOP1. (b) Amino acid sequence alignment of STOP1 from strawberry and other plant species. The sequences for alignment were the 255–417 aa region of the FvSTOP1. The amino acid sequence alignment was done using DNAMAN software. The red triangles represent the location of the zinc finger domains. Figure S2 The leaves and flowers phenotypes of overexpressing and knockout FvSTOP1 transgenic strawberry plants. (a) The leaves and flowers phenotypes of wild‐type (‘Ruegen’, WT) and overexpressing FvSTOP1 plants (FvSTOP1‐OE). (b) The leaves and flowers phenotype of WT and FvSTOP1 knockout transgenic strawberry plants (fvstop1‐cr). Scale bar = 1 cm. Figure S3 Gene editing types of FvSTOP1 knockout transgenic strawberry plants. sgRNA1 and sgRNA2 target sites were mutated in fvstop1‐1, fvstop1‐2, and fvstop1‐3 strawberry plants. The dotted red line is the sgRNA position. The ellipsis represents the missing nucleotides. The red font indicates the inserted nucleotide. The yellow box is the intermediate sequence between sgRNA1 and sgRNA2. (a) The gene editing type of fvstop1‐1 line (a 295 bp base inversion in the interval of sgRNA1 and sgRNA2). (b) Sequencing diagram of fvstop1‐1. (c) fvstop1‐2 is a cross of fvstop1‐1 and fvstop1‐3. (d) Sequencing diagram of fvstop1‐2. (e) fvstop1‐3 line with a T nucleotide inserted at sgRNA1 to form the stop codon TGA and a nucleotide missing at sgRNA2. (f) Sequencing diagram of fvstop1‐3. Figure S4 The developmental phenotypes of WT and fvstop1‐cr 3# transgenic strawberry plants. The phenotypes of WT and fvstop1‐cr 3# transgenic strawberry plants at 30, 60, and 90 days after transplanting to the greenhouse. Scale bar = 1 cm. Figure S5 Flavonoid metabolomics analysis of WT a [file PBI-23-3727-s002.docx]

**Supplemental information**

**Supplemental Figure**

**
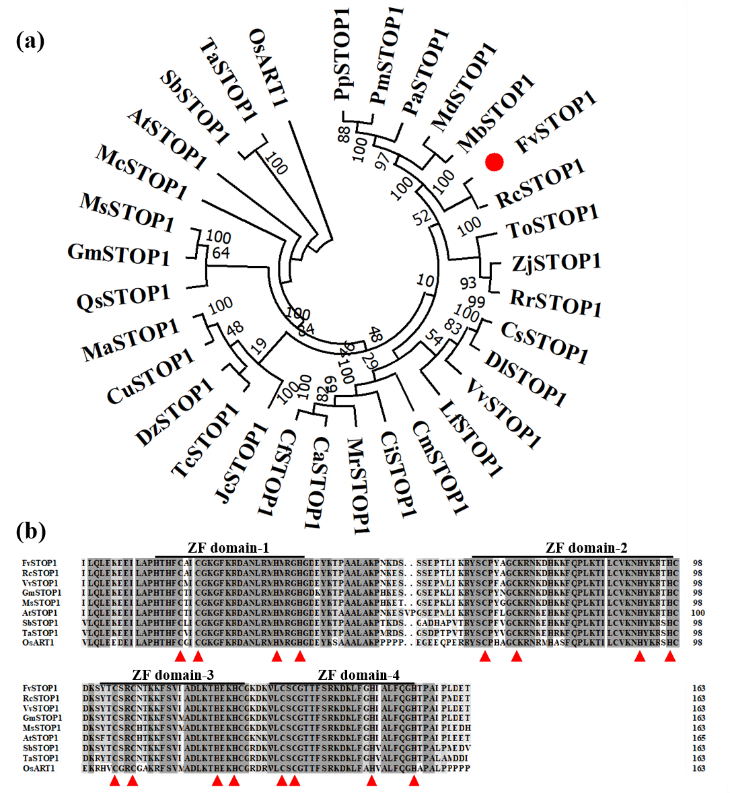
**

**Supplemental Figure S1.** **Phylogenetic and structural analyses of FvSTOP1 in strawberry.**

(a) Phylogenetic relationship of protein sequences of STOP1 from strawberry and other plant species. The phylogenetic analysis was aligned using the neighbor-joining method. The red circle represents FvSTOP1. (b) Amino acid sequence alignment of STOP1 from strawberry and other plant species. The sequences for alignment were the 255-417aa region of the FvSTOP1. The amino acid sequence alignment was done using DNAMAN software. The red triangles represent the location of the zinc finger domains.

**
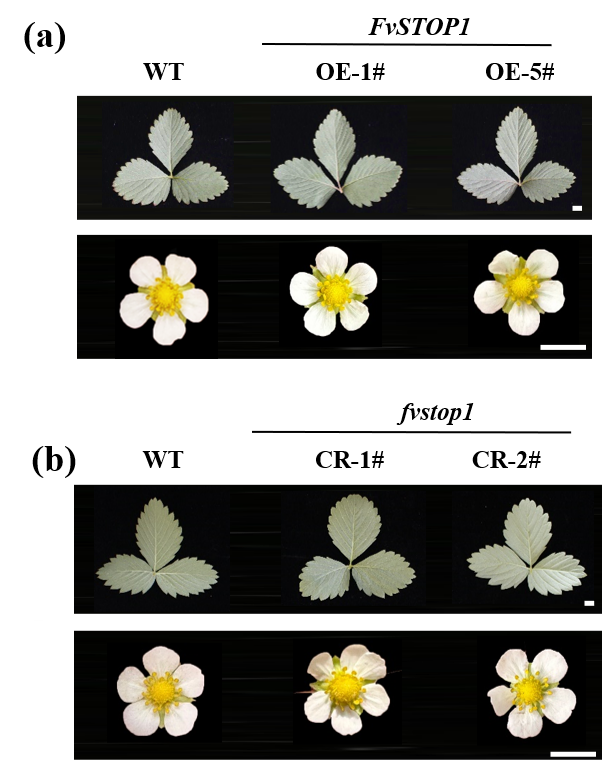
**

**Supplemental Figure S2.** **The leaves and flowers phenotypes of overexpressing and knockout *FvSTOP1* transgenic strawberry plants.**

(a) The leaves and flowers phenotypes of wild-type (‘Ruegen’, WT) and overexpressing *FvSTOP1* plants (FvSTOP1-OE). (b) The leaves and flowers phenotype of WT and *FvSTOP1* knockout transgenic strawberry plants (*fvstop1*-*cr*). Scale bar = 1cm.

**
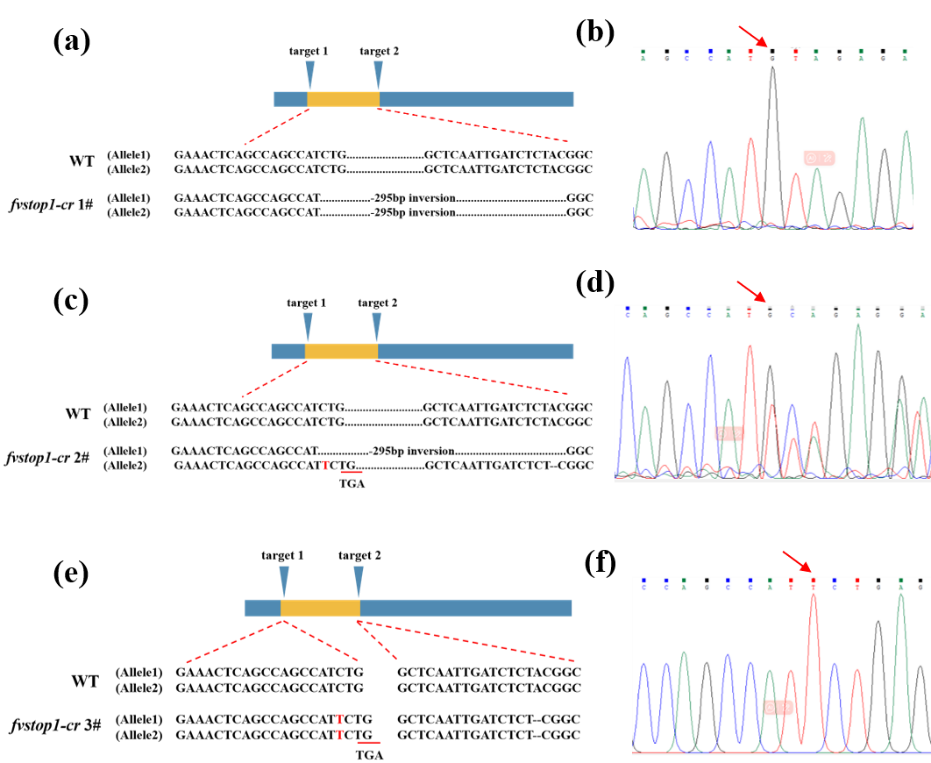
**

**Supplemental Figure S3.** **Gene editing types of *FvSTOP1* knockout transgenic strawberry plants.**

sgRNA1 and sgRNA2 target sites were mutated in *fvstop1-1*, *fvstop1-2*, and *fvstop1-3* strawberry plants. The dotted red line is the sgRNA position. The ellipsis represents the missing nucleotides. The red font indicates the inserted nucleotide. The yellow box is the intermediate sequence between sgRNA1 and sgRNA2. (a) The gene editing type of *fvstop1-1* line (a 295bp base inversion in the interval of sgRNA1 and sgRNA2). (b) Sequencing diagram of *fvstop1-1.* (c) *fvstop1-2* is a cross of *fvstop1-1* and *fvstop1-3.* (d) Sequencing diagram of *fvstop1-2.* (e) *fvstop1-3* line with a T nucleotide inserted at sgRNA1 to form the stop codon TGA and a nucleotide missing at sgRNA2.  (f) Sequencing diagram of *fvstop1-3.*


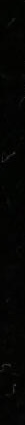

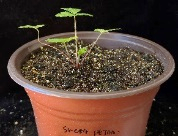

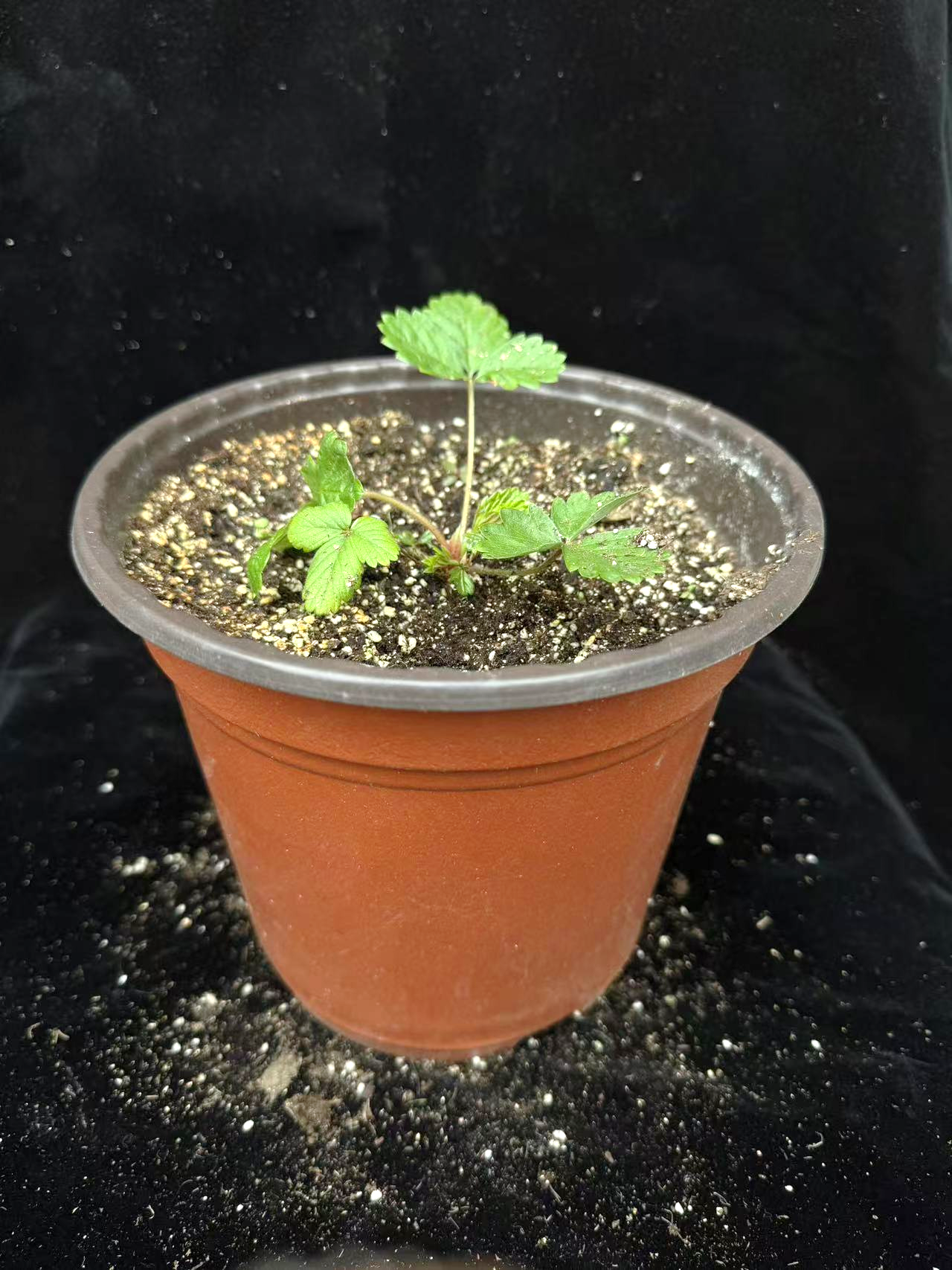

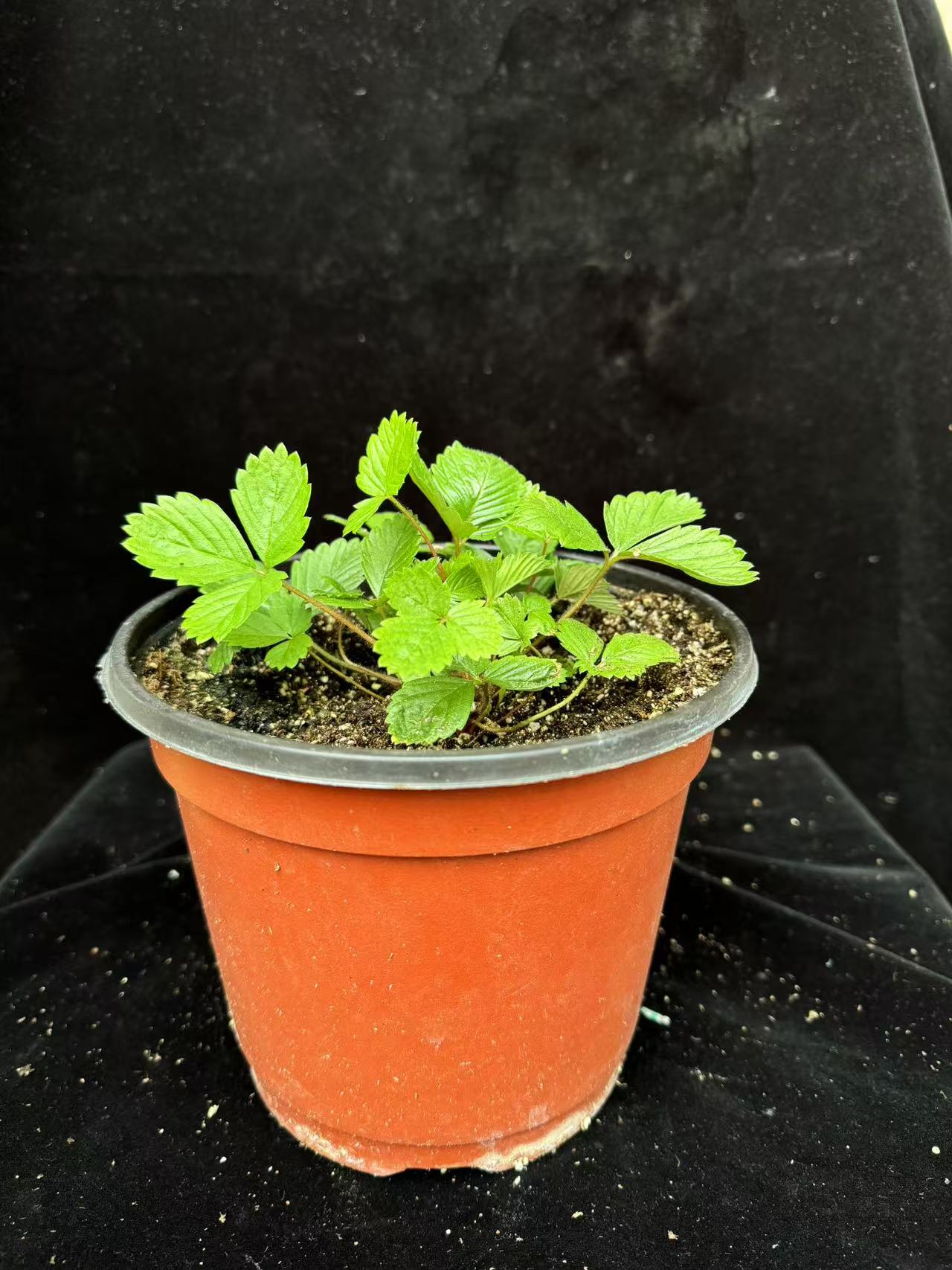

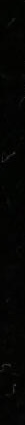

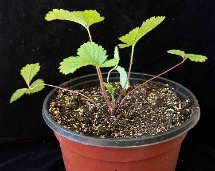

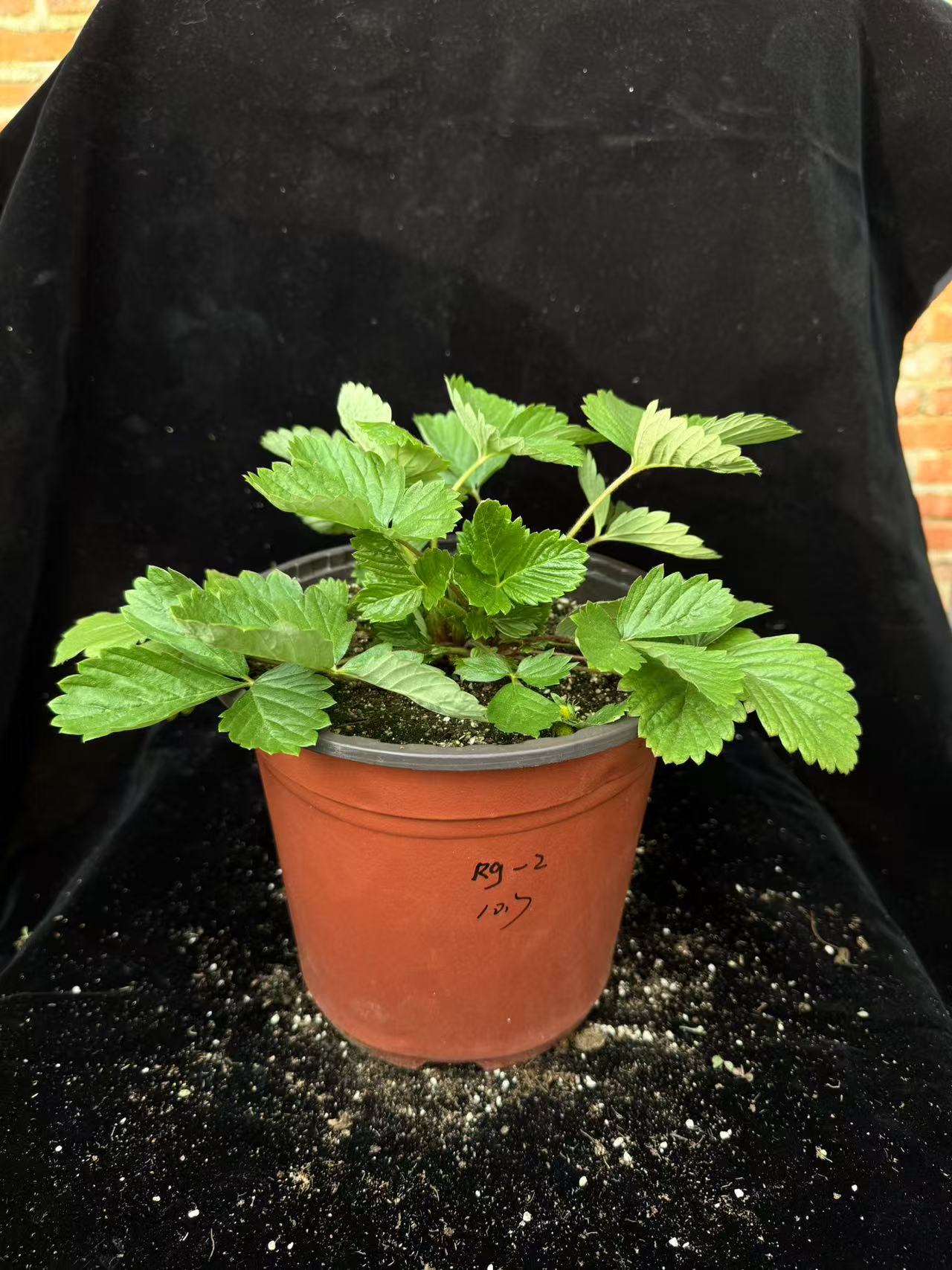

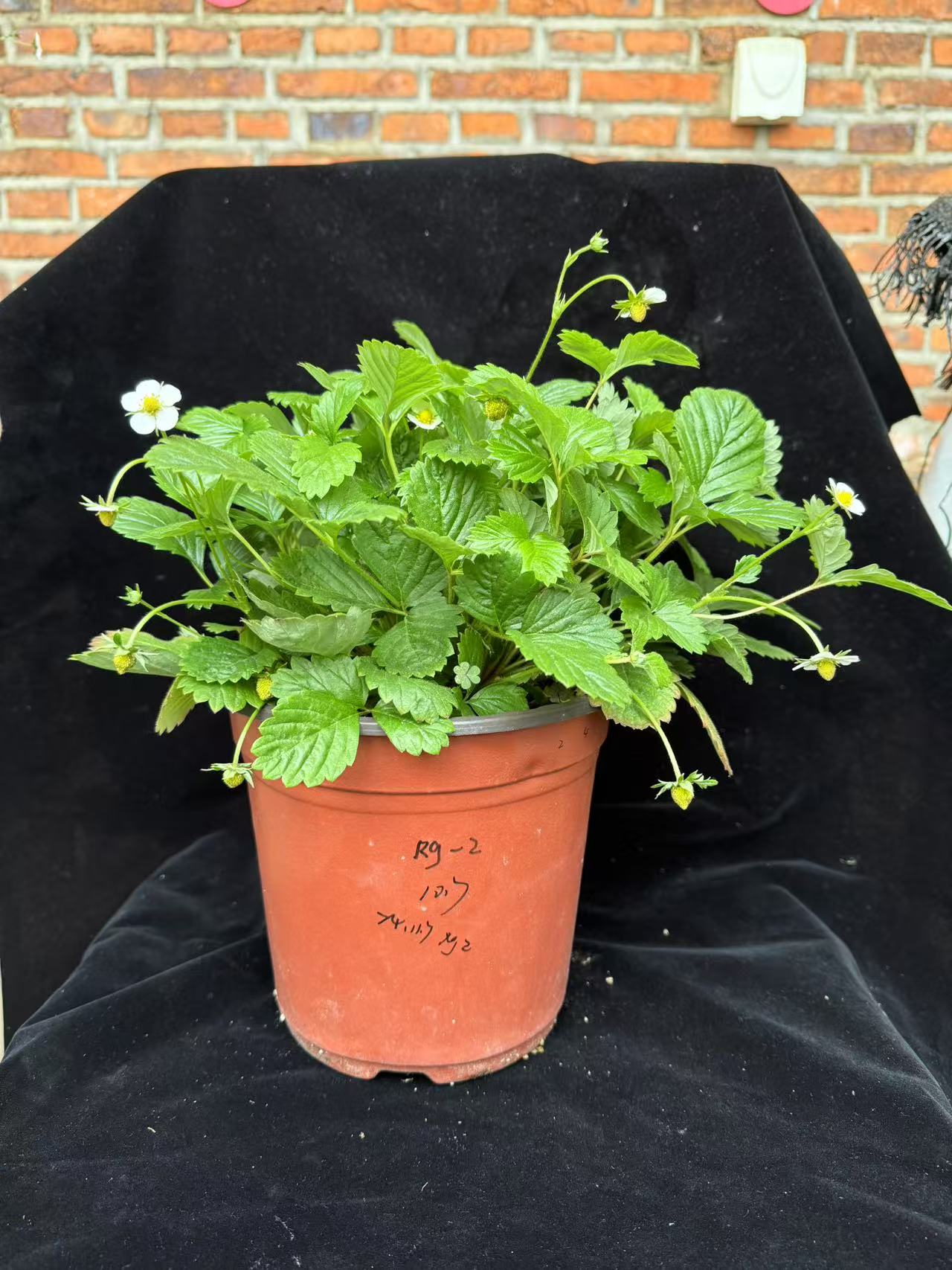


**DPA 30 60 90**

**WT**

***fvstop1-cr 3#***

**Supplemental Figure S4. The developmental phenotypes of WT and *fvstop1-cr* 3# transgenic strawberry plants.**

The phenotypes of WT and *fvstop1-cr* 3# transgenic strawberry plants at 30, 60, and 90 days after transplanting to the greenhouse. Scale bar = 1cm.


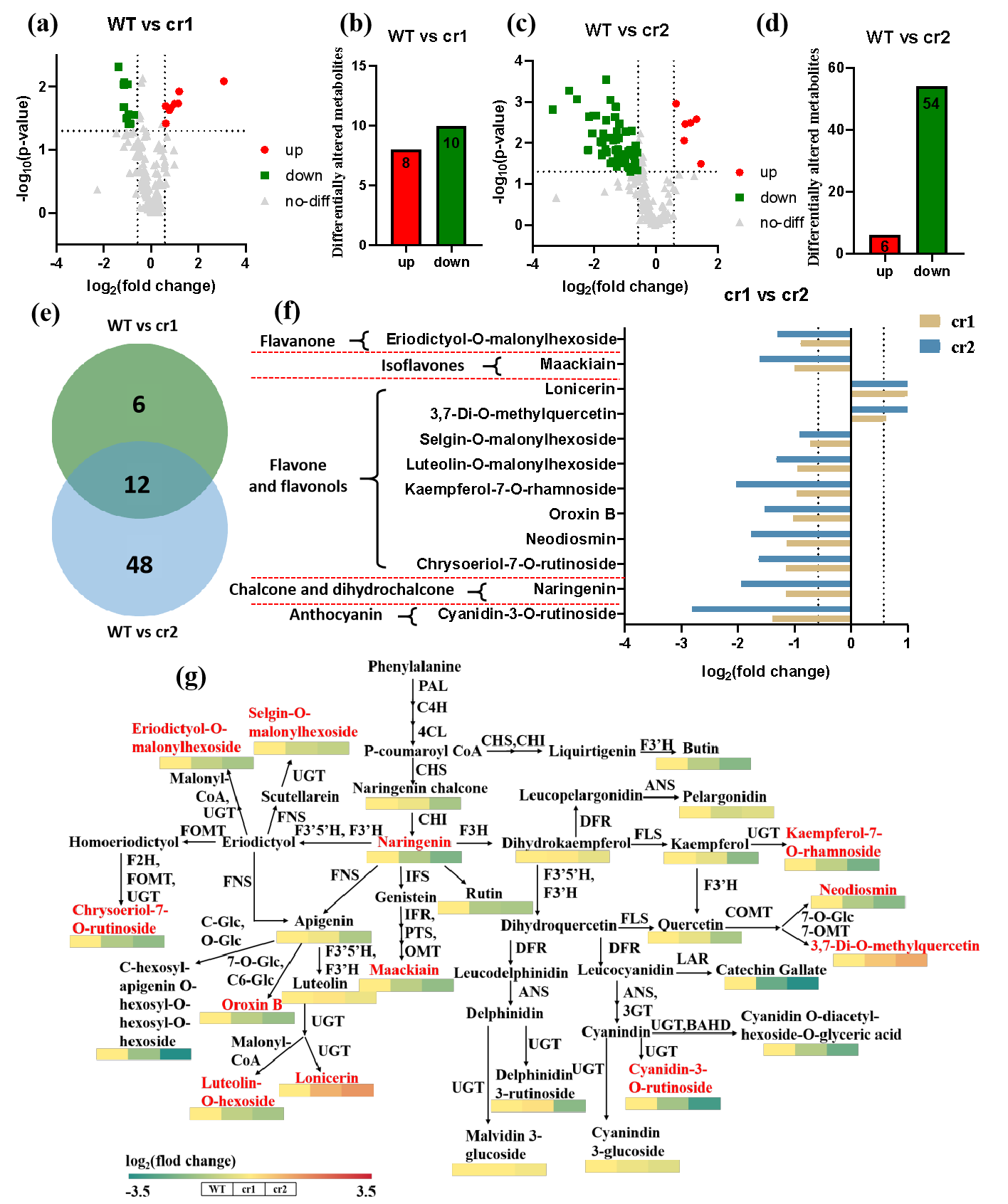


**Supplemental Figure S5.** **Flavonoid metabolomics analysis of WT and *fvstop1-cr* 1#, *fvstop1-cr* 2# knockout transgenic strawberry plants.**

(a) Volcano diagram of metabolic profiles in WT and *fvstop1-cr* 1#. The red circles, green squares, and gray triangles represent the up-regulated, down-regulated, and insignificant metabolites, respectively. The horizontal axis represents the fold change of the metabolites content, and the vertical axis represents the significant level of the difference. (p-value < 0.05 and |log_2_FC|>0.58). (b) Differential altered metabolites in WT and *fvstop1-cr* 1#. (c) Volcano diagram of metabolic profiles in WT and *fvstop1-cr* 2#. The red circles, green squares, and gray triangles represent the up-regulated, down-regulated, and insignificant metabolites, respectively. The horizontal axis represents the fold change of the metabolites content, and the vertical axis represents the significant level of the difference. (p-value < 0.05 and |log_2_FC|>0.58). (d) Differential altered metabolites in WT and *fvstop1-cr* 2#. (e) Differential altered metabolites of WT vs *fvstop1-cr* 1# and WT vs *fvstop1-cr* 2# on the Venn diagram. (f) Commonly shared down-regulated metabolites of WT vs *fvstop1-cr* 1# and WT vs *fvstop1-cr* 2#. (p-value < 0.05 and |log_2_FC|>0.58). (g) Simpliﬁed scheme of flavonoid metabolic pathway in plants. The metabolites that were significantly regulated in *fvstop1-cr* 1# and *fvstop1-cr* 2# compared with WT are indicated in red font. Square visually represents the altered content levels of differential metabolites. (p-value < 0.05 and |log_2_FC|>0.58). PAL: phenylalanine ammonia lyase, C4H: cinnamate-4-hydroxylase, 4CL: 4-coumarate CoA ligase, CHS: chalcone synthase, CHI: chalcone isomerase, F3H: ﬂavanone 3-hydroxylase, F3’H: ﬂavonoid 3’-hydroxylase, F3’5’H: flavonoid-3’,5’-hydroxylas, DFR: dihydroﬂavonol 4-reductase, ANS: anthocyanidin synthase, FNS: flavone synthase, FLS: flavonol synthase, LAR: leucoanthocyanidin reductase, F2H: flavanone-2-hydroxylase, IFR: isoflavone reductase, PTS: pterocarpan synthase, IFS: isoflavone synthase, OMT: O-methyltransferase, FOMT: flavonoid O-methyltransferase, COMT: catechol-O-methyltransferase, 3GT: flavonoid-3-O-glucosyltransferase, UGT: UDP-ﬂavonoid glucosyl transferase, BAHD: acyl-CoA dependent acyltransferases, O-Glc: O-linked β-N-acetylglucosamine, C-Glc: C-linked glycosylation.

**
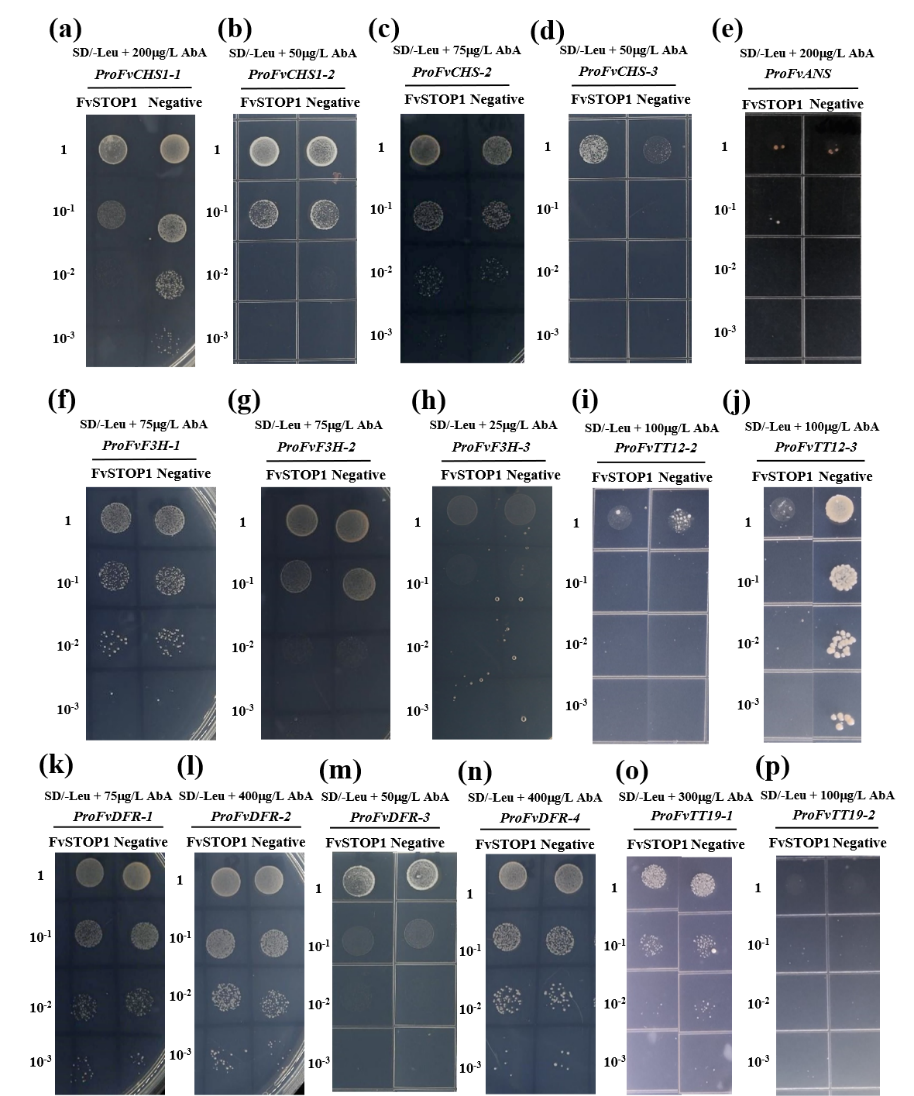
**

**Supplemental Figure S6.** **FvSTOP1 cannot bind to the promoters of*FvCHS*, *FvF3H*, *FvDFR*, *FvANS*, and *FvTT12* by yeast one-hybrid (Y1H) assay.**

*FvCHS*, *FvF3H*, *FvDFR*, *FvANS*, and *FvTT12* promoter regions were segmented and ligated to the pAbAi vector. The pGADT7 vector was the control. The plasmids harboring promoters-pAbAi and FvSTOP1-AD were transferred into Y1H yeast cells. Positive single colonies were selected and assigned to SD/−Leu and SD/-Leu/+AbA (25-500 μg/L) medium.  (a-d) FvSTOP1 cannot bind to the promoters of*FvCHS.* (e) FvSTOP1 cannot bind to the promoters of*FvANS.* (f-h) FvSTOP1 cannot bind to the promoters of*FvF3H*. (i-j) FvSTOP1 cannot bind to the promoters of*FvTT12.* (k-n) FvSTOP1 cannot bind to the promoters of*FvDFR.* (o-p) FvSTOP1 cannot bind to the promoters of*FvTT19-1* and*FvTT19-2.* The segmentations are as follows: *proFvCHS*(*proFvCHS1-1*: -1790--1548bp,*proFvCHS1-2*: -857--568bp,*proFvCHS2*: -1570--836bp，*proFvCHS3*: -588--1bp), *proFvF3H* (*proFvF3H-1*: -2001--1032bp, *proFvF3H-2*: -1091---608bp *proFvF3H-3*: -646--1bp), *proFvDFR* (*proFvDFR-1*: -2253--1638bp,*proFvDFR-2*: -1698—1042bp, *proFvDFR-3*: -1113--463bp,*proFvDFR-4*: -552--1bp), *proFvANS*(-2001--1bp),*proFvTT12* (*proFvTT12-2*: -1056--481bp,*proFvTT12-3*: -552--1bp),*proFvTT19* (*proFvTT19-1*: -1455--913bp *, proFvTT19-2*: -975--479bp*, proFvTT19-3*:-534--1bp).

**Supplemental Figure S7.** **The expression patterns of *FvTT19*in different organs of woodland strawberry.**

RT-qPCR detected the expression patterns of *FvTT19* in different organs of woodland strawberry. Different letters indicated significant differences compared with the shoot apex. Data are the mean ±SD of three biological replicates with Tukey's hoc test (P < 0.05).

**
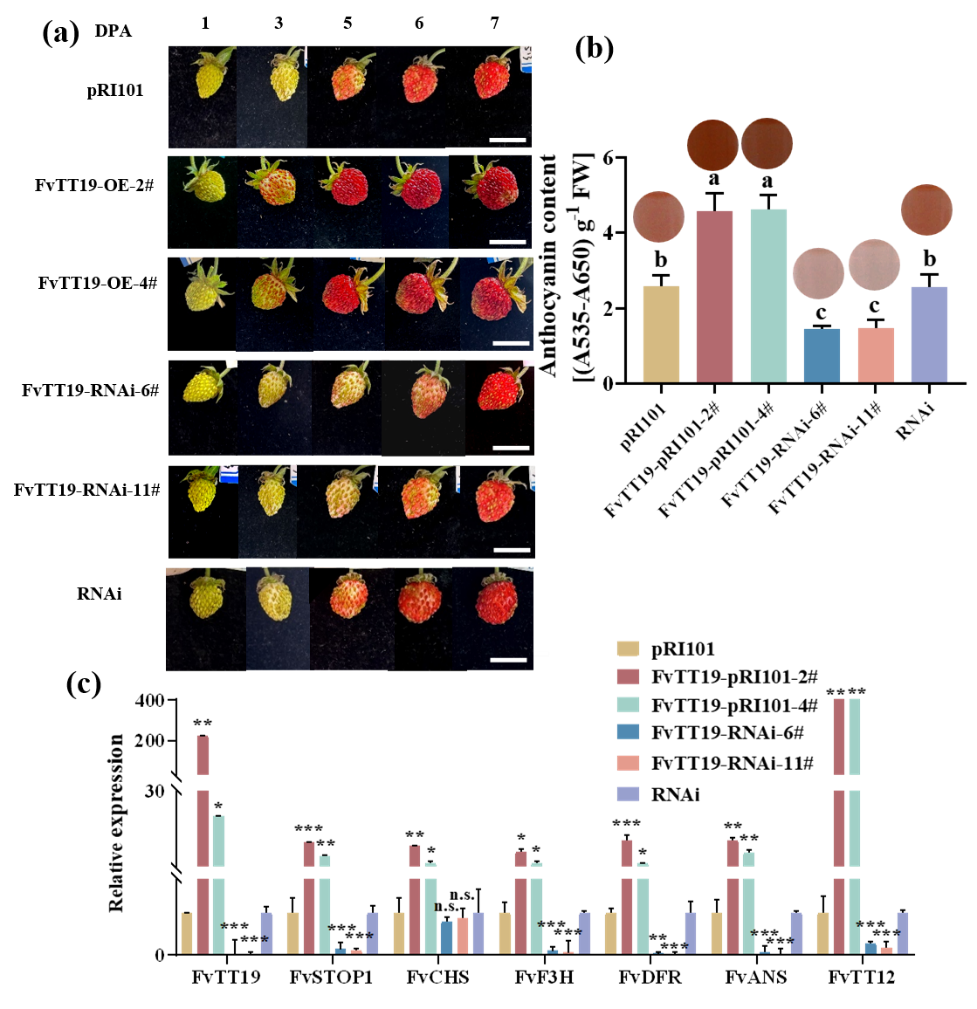
**

**Supplemental Figure S8.** **Functional analysis of *FvTT19* in strawberry fruits by transient expression assay.**

(a) The Agrobacterium bacterial suspension harboring *p35S*::*FvTT19* and *FvTT19-*RNAi were constructed and injected into the green stage fruits (18 days after pollination) of the ‘Ruegen’, and at least 15 fruits (green stage) were selected for transient transformation. Scale bar = 1cm. (b) Anthocyanin contents in transient transformation fruits. The circles represent the color of the anthocyanin extract. FW is Fresh weight. Different letters represent significant differences compared to the control with Tukey's hoc test (P < 0.05). (c) Relative expression of anthocyanin-related genes in*p35S*::*FvTT19* and *FvTT19-*RNAi transient transformation fruits. Data are the mean ±SD of three biological replicates. Significant differences were analyzed by Student’s t-test (*P < 0.05，**P < 0.01，***P < 0.001).

**(a)**

**(b)**

**Supplemental Figure S9.** **Relative expression of anthocyanin-related genes in transient transformation fruits with different combinations.**

(a) Relative expression of anthocyanin-related genes in co-injection of FvMYB1-RNAi and FvbHLH33-RNAi plasmids into the green stage fruits (18 days after pollination) of the ‘Ruegen’. (b) Relative expression levels of anthocyanin-related genes in co-injection of FvMYB1-RNAi and FvbHLH33-RNAi plasmids into the green stage fruits of the ‘Ruegen’ and *fvstop1-cr* 2# plants. Data are the mean ±SD of three biological replicates. Significant differences were analyzed by Student’s t-test (*P < 0.05，**P < 0.01，***P < 0.001).

**Supplemental Figure S10.** **The coding sequence alignment between *FvTT19* and*RAP*.**
